# ABAG-Rank: improving model selection of AlphaFold antibody–antigen complexes by learning to rank

**DOI:** 10.1093/bioinformatics/btag663

**Published:** 2026-09-04

**Authors:** Matteo Tadiello, Marko Ludaic, Vsevolod Viliuga, Arne Elofsson

**Affiliations:** Department of biochemistry and biophysics (DBB) and SciLifeLab, Stockholm University, Stockholm, 171 65, Sweden; Department of biochemistry and biophysics (DBB) and SciLifeLab, Stockholm University, Stockholm, 171 65, Sweden; Department of biochemistry and biophysics (DBB) and SciLifeLab, Stockholm University, Stockholm, 171 65, Sweden; Max Planck Institute for Polymer Research (MPIP), Mainz, 55128, Germany; Department of biochemistry and biophysics (DBB) and SciLifeLab, Stockholm University, Stockholm, 171 65, Sweden

## Abstract

**Motivation:**

AlphaFold has transformed structural biology with an unprecedented accuracy in modelling protein structures and their interactions with biomolecules, with AlphaFold3 (AF3) achieving state-of-the-art performance. However, AF3 and other methods often struggle to accurately predict the structure of protein complexes that lack strong co-evolutionary information, such as antibody–antigen (Ab–Ag) complexes. One of the fundamental issues is that AF3 often generates accurate predictions, but fails to reliably distinguish them from the much larger set of incorrect ones.

**Results:**

To address this, we propose ABAG-Rank, a deep neural network that provides an efficient and robust solution for model selection of Ab–Ag interactions from a pool of structural ensembles predicted with AlphaFold. Built on the permutation-invariant DeepSets architecture, ABAG-Rank can process variable-sized ensembles of structural decoys and is directly applicable to prediction settings in which the number of candidates may vary. We train a model on a redundancy-reduced set of all known antibody–antigen complexes and find that simple geometric descriptors, along with confidence scores from AlphaFold, provide rich information about interface quality without requiring intensive physics-based calculations. Our experiments demonstrate that ABAG-Rank significantly outperforms AF3 internal scoring and the ranking performance of existing deep learning baselines.

**Availability and Implementation:**

Source code can be found at: https://github.com/tadteo/ABAG-Rank or on Zenodo at https://doi.org/10.5281/zenodo.21132090

## 1 Introduction

The release of AlphaFold2 (AF2) (Jumper *et al.* 2021) has truly revolutionized the field of structural biology. By achieving unprecedented accuracy in predicting the three-dimensional structure of proteins from their one-dimensional amino acid sequences, AF2 has become a standard tool in almost all structural studies. Building on the foundations and best practices of AF2, AlphaFold3 (AF3) (Abramson *et al.* 2024) was extended to model all-atom protein structures in complexes with metal ions, nucleic acids, small-molecule ligands, and other biomolecules. This computational breakthrough enabled researchers to infer protein functions and interactions prior to experimental validation, substantially reducing exploratory laboratory costs and efforts. However, accurately predicting molecular interfaces remains a grand challenge, particularly for complexes in which highly specific binding interactions determine recognition and affinity, and distinct co-evolutionary information may be too scarce or absent altogether.

**Figure 1:**
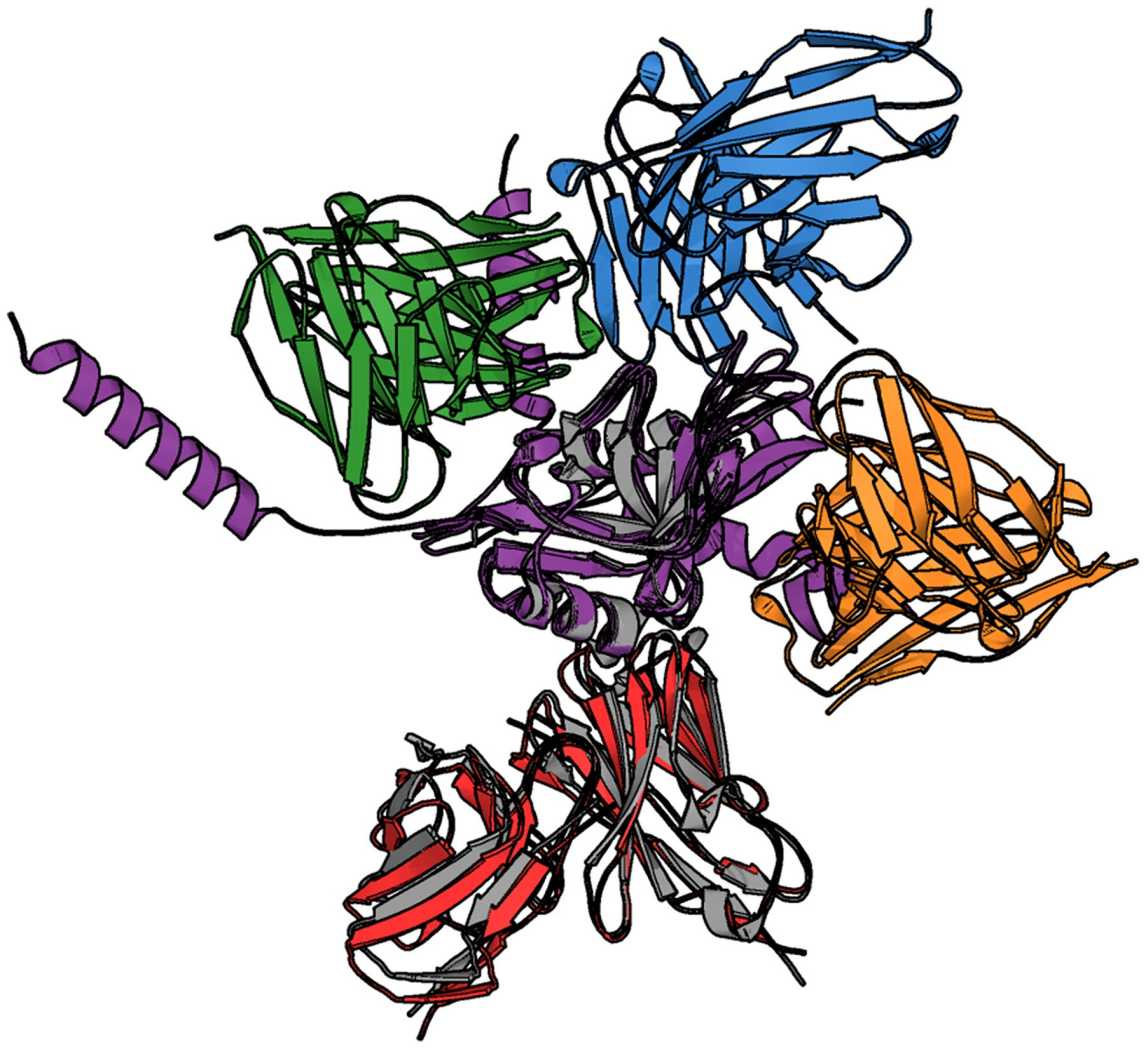
Structural diversity of AlphaFold3 predictions for an example antibody–antigen complex (PDB ID: 8URF). AlphaFold3 frequently places the antibody CDR loops at distinct, non-overlapping interfaces on the antigen. Although many of these binding modes might appear geometrically plausible, they are often biologically incorrect. The antigen is shown in purple, the reference ground-truth structure in grey, and different predicted antibody poses in green, blue, orange, and red.

Despite their overall high accuracy, the quality of the predictions produced with AF2/3 critically depends on the model’s internal confidence metrics, namely the predicted TM-score (pTM) and interface pTM (ipTM) scores (Jumper *et al.* 2021). These scores are used to rank candidate structures based on their predicted reliability. These internal ranking metrics, however, although they provide a broadly informative signal, often fail to adequately distinguish between distinct predictions, overestimating the quality of models that incorrectly predict molecular interfaces, especially for specific subsets of proteins, such as antibody–antigen (Ab–Ag) complexes, as shown in (Fromm *et al.* 2025, Smorodina *et al.* 2026). Unlike general protein–protein interactions, Ab–Ag complexes stand out with their unique structural and functional complexity and the lack of co-evolutionary information (Gao and Skolnick 2024). Antibody binding is mediated by hypervariable loops (Complementarity-Determining Regions, CDRs), and particularly the H3 loop, which exhibits immense sequence diversity and conformational flexibility, obscuring the strong structural prior needed for confident prediction. One of the most common issues is the placement of antibody chains at incorrect binding sites (epitopes) (Gao and Skolnick 2024, Fromm *et al.* 2025). AlphaFold often generates decoys in which the antibody is docked to the wrong epitope on the antigen—a region that may exhibit high geometric complementarity but in reality lacks biological relevance (Fig. 1). These incorrect binding modes can nevertheless receive high pTM/ipTM confidence scores, distorting the ranking and effectively hallucinating a high-confidence interaction at an incorrect binding site. Consequently, binding poses that more closely align with the ground-truth epitope can be overlooked if AF incorrectly scores them as lower-quality than these high-scoring false positives.

One of the most established strategies for antibody–antigen structure prediction is extensive sampling; this approach trades computational cost for a higher probability of accurate predictions. AFsample (Wallner 2023), for instance, applies aggressive dropout-based stochastic inference with AF2 to generate large and diverse structural ensembles per protein complex, while MassiveFold (Raouraoua *et al.* 2024) extends this strategy to large-scale computational clusters, significantly reducing computation time from days to hours. However, the persistence of unreliable confidence-based ranking renders ranking fidelity a fundamental limitation, such that even with aggressive sampling strategies, structurally more accurate solutions may still be overlooked if they are assigned lower confidence scores.

To improve the heuristic confidence estimates of AF2/3, several alternative interface quality metrics have been proposed. For example, pDockQ (Bryant *et al.* 2022) and pDockQ2 (Zhu *et al.* 2023) directly use PAE and pLDDT outputs of AF2 and map them to DockQ-like (Basu and Wallner 2016) scores through a simple sigmoid fit, resulting in improved interface quality estimation for protein complexes. Other approaches, such as actifpTM (Varga *et al.* 2025) and ipSAE (Dunbrack 2025), modify (i)pTM and PAE more explicitly: actifpTM reweights residue pairs using predicted distance probabilities such that interacting residues contribute more strongly than non-contacting ones, thereby reducing length-dependent bias from non-interacting regions. ipSAE, in turn, restricts the calculation to inter-chain residue pairs with reliably low PAE and rescales the score accordingly to reduce the inflated confidence. Despite the refinements, such purely AF-confidence-based alternatives remain conceptually limited in their generalizability, particularly for antibody–antigen complexes, where AlphaFold confidence metrics alone are often insufficient to distinguish correct from incorrect binding modes. Alternatively, several deep learning-based interface scoring approaches, such as EuDockScore (McFee *et al.* 2024) and GDockScore (McFee and Kim 2023) have been proposed. However, their applicability to antibody–antigen complexes remains unclear, as they have not been systematically benchmarked in this setting. The only scoring model specifically developed for Ab–Ag interactions is the recently released DeepRank-Ab (Xu *et al.* 2026), an equivariant graph neural network that predicts DockQ from a large set of biophysics-based interface descriptors. Because these features must be computed with HADDOCK (Giulini *et al.* 2025), the method introduces external dependencies and is comparatively inefficient.

This work proposes **ABAG-Rank—**a deep neural network trained to rank the quality of Ab–Ag predictions from AlphaFold-like models and designed to address the limitations of both heuristic AlphaFold-derived confidence scores and existing deep learning-based interface scoring approaches. Leveraging the DeepSet architecture (Zaheer *et al.* 2017), the model treats each decoy ensemble as a permutation-invariant, variable-sized set rather than as a collection of isolated instances, which is well suited to the practical setting where the number of predictions per complex varies and the structural diversity within an ensemble can range from highly heterogeneous to nearly collapsed around a single structural solution. By integrating regression and ranking objectives into a composite loss, ABAG-Rank is trained to resolve subtle quality differences between alternative binding modes within the same Ab–Ag complex, even in the presence of highly skewed DockQ distributions and strong complex-to-complex variability. In addition, we introduce a redundancy-reduced dataset generation strategy and show that a smaller but statistically diverse training set is sufficient to learn a robust ranking model. Our experiments show that AbAb-Rank consistently outperforms both AF3 and DeepRank-Ab in ranking interface quality and in retrieving the highest-quality models from predicted structural ensembles. We further demonstrate that AF3-derived confidence scores, particularly iPTM and inter-chain Predicted Aligned Error (PAE), together with pairwise interface distances and ESM2 protein language model embeddings (Lin *et al.* 2023), provide an informative basis for accurate assessment of interface quality.

## 2 Method

Below, we describe the dataset construction, model architecture, and the core design choices underlying ABAG-Rank. To train a model to rank the quality of Ab–Ag complexes, we first generate a diverse dataset of Ab–Ag models using AF3 predictions. Second, we develop a dedicated ranking component that complements the native AF3 confidence metrics. AF3 can produce multiple models for a given complex by varying the random seed or the number of diffusion timesteps. Our goal is to provide an interface quality predictor that reliably ranks models, especially when multiple predictions for the same complex are available, and to improve the model’s ability to distinguish good from bad interfaces.

### 2.1 Dataset preparation

Using the Structural Antibody Database (SAbDab) (Dunbar *et al.* 2014) as our initial source of antibody–antigen complexes, we constructed an Ab–Ag dataset designed to maximize diversity in interface quality and Predicted Aligned Error (PAE) matrices as well as structural diversity. We used data points (structure decoys) for training and testing, applying a cutoff-date approach as in AlphaFold. Using the following split:

- **Training:** all SAbDab entries till 2024/09/30.
- **Test:** SAbDab entries from 2024/09/30 to 2025/06/01.

This allow us to have AF3 predictions both from and outside it’s training set, having a large enough dataset for training enough variability in AF3 confidence, necessary for the model to have samples of diverse quality. An analysis of prediction distribution of AF3 vs ABAG-Rank can be seen in S20 and S18. For each complex, we run inference with AF3 using 10 random seeds and generate in total 50 structural decoys (5 samples per seed). For each of the decoys, we retrieve the full N×N Predicted Aligned Error (PAE) matrix (*N*: number of residues in a complex), along with the model’s internal confidence scores: pTM and ipTM, and the composite ranking score. To capture explicit geometric constraints, we compute pairwise Cα distance matrices and derive per-residue features from them (i.e. each residue’s distance to all other residues). Since our focus is to quantify the interface quality, special attention is paid to inter-chain interactions; indices corresponding to antibody–antigen interface residues are extracted to emphasize the model’s focus on the binding interface.

To generate ground-truth labels for training, we compare each generated decoy with experimentally determined reference structures in the SabDab database. Given that antigens may comprise multiple chains, we implement a logical chain-merging step prior to scoring. All antigen chains are merged into a single “Ag” entity, and antibody heavy/light chains are merged into an “Ab” entity. The structural quality is then assessed using DockQ (Basu and Wallner 2016).

Decoys where metric computation fails (e.g. due to alignment timeouts or missing chains) are filtered out to maintain dataset integrity.

Prior to redundancy reduction, we compute ensemble-level statistics, namely the column-wise mean, median, and standard deviation of the inter-chain PAE and $C_{\alpha }$ distances, across all generated decoys. These global context features are calculated on the full, unfiltered ensemble to ensure they accurately reflect the aleatoric uncertainty of AF3 predictions. Preservation of these statistics allows the model to assess an individual decoy’s confidence relative to the true population distribution, even after redundant samples are pruned.

We define redundancy based on three criteria: structural quality similarity (DockQ and RMSD) and confidence profile similarity (PAE distribution). First, we establish an adaptive PAE similarity threshold, $\tau_{\text{PAE}}$, for each complex. This is derived by randomly sampling 500 decoy pairs, computing the Wasserstein distance between their flattened PAE vectors, and setting $\tau_{\text{PAE}}$ to the 25th percentile of these distances. The DockQ difference threshold is fixed at $\tau_{\text{DockQ}}=0.01$ the RMSD difference threshold is fixed at $\tau_{\text{RMSD}}=$ 4Å.

We then apply a greedy pruning algorithm to cluster similar decoys in the training set (Algorithm 2 and 1 are provided in the Supplementary Material). A sample is considered redundant and removed only if it is sufficiently similar to an already selected sample in terms of PAE distribution (Wasserstein distance $<\tau_{\text{PAE}}$) *and* has a nearly identical DockQ score (difference $<\tau_{\text{DockQ}}$) and the RMSD of the sample is $<\tau_{\text{RMSD}}$. This ensures that we retain multiple conformations if they differ significantly in either geometry or model confidence. The overall distribution of real DockQ remains the same but drastically reducing the number of samples S19.

The final dataset contains 4610 distinct complexes in the training and validation sets, with an average of 20 distinct samples per complex (after filtering), and 1091 complexes in the test set, with exactly 50 samples per complex.

### 2.2 Model architecture and nested complex batching

**Figure 2:**
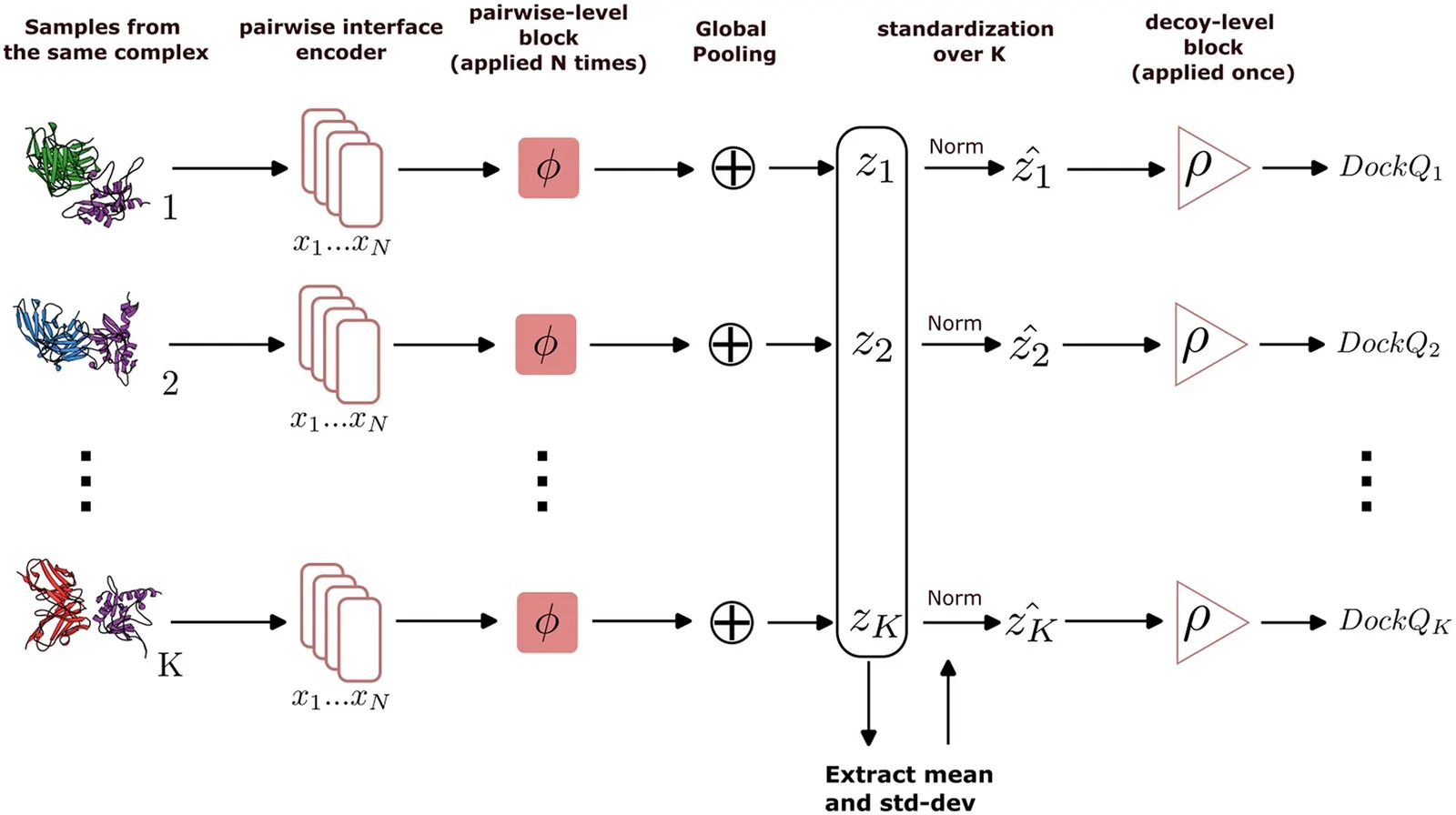
ABAG-Rank Architecture. The model processes a variable-sized set of *K* decoys per complex. For each decoy, inter-chain residue pairs are encoded with geometric features (PAE, Distance, Residue type) and optionally evolutionary context (ESM embeddings) (Lin *et al*. 2023). These pair features are processed by a shared encoder φ and aggregated via concatenated statistics (Mean, Max, Attention, interface-length). The resulting global descriptor is standardized over the K samples for complex b. The normalized embedding is passed to a decoder ρ to predict the interface quality score.

We adopt a DeepSet-inspired (Zaheer *et al.* 2017) architecture designed to process variable-sized sets of inter-chain residue pairs while preserving permutation invariance Fig. 2. The permutation-invariant architecture is essential for treating the interface features of Ab–Ag complexes as a set of individual pairs, focusing on distance and properties while preventing the model from picking up unwanted biases stemming from residue ordering. Moreover, this architecture dynamically handles *N* pairs. By using global pooling, we collapse any number of residue-level features into a fixed-length “complex-level” embedding. This allows us to score any complex, regardless of size. Moreover, PAE is inherently asymmetric; for this reason, (i,j) and (j,i) pairs have different values and both are processed by the model. In contrast to standard pooling, we employ an aggregation strategy that combines multiple permutation-invariant statistics with a global attention pooling head, allowing the model to capture both the average interface quality and sparse structural outliers within an interface. We restrict the set of pairs to those within a spatial cutoff of 12Å (based on inter-chain Cα distance), treating pairs beyond this cutoff as non-interacting and thus excluded from the interface representation.

We propose a nested per complex batching. For each complex in the batch *B*, a nested tensor is computed by sampling *K* decoys for each complex *b* independently. Each training sample has hence the shape $X\in R^{B\times K\times N\times F}$ to reflect the underlying *set-of-decoys per complex* structure unique of the ranking problem, where *B* is the batch size, *K* is the number of decoys sampled per complex, *N* is the number of inter-chain residue pairs and *F* the number of features per residue pair. This helps to differentiate decoys from the same complex population. This hierarchical batching strategy encourages the network to learn *within-complex* relative ordering: the model observes diverse conformations in the same antibody–antigen context. This step is essential for the model to enforce the capability to differentiate interfaces for predictions of the same complex *b* using the information shred by each decoy *k*.

### 2.3 Model input features

We hypothesise that much of the information required for accurate interface ranking is already implicitly encoded in (i) geometric features of the predicted interface and (ii) residue-level evolutionary signals captured by protein language model embeddings. Inter-chain $C_{\alpha }$ distances provide a direct description of geometric complementarity, while residue type encodings and ESM2 embeddings supply some learned biochemical context that can help distinguish biologically meaningful interactions from geometrically plausible artefacts. We further expect AlphaFold3 internal confidence scores to provide complementary information about the reliability of the predicted interface. Because this feature set explicitly depends on AlphaFold-derived outputs (PAE, pTM, ipTM), ABAG-Rank is designed for AF-like structure predictors and is not directly applicable to decoys generated by conventional docking methods, which do not provide comparable confidence outputs. Given the permutation-invariant architecture, positional encodings are included to restore sequential pair-specific information that would otherwise be lost in set-based processing.

Hence, for each residue pair (i,j) we construct a feature vector $x_{ij}\in R^{F}$ comprising:

- inter-chain Cα Euclidean distance;
- inter-chain predicted aligned error (PAE), centred by the complex-level mean;
- complex-specific mean interface PAE (global context);
- positional encoding based on a Cantor pairing of indices (i,j), normalized to [0,1];
- residue type encoding (e.g. one-hot identity and/or ESM2-derived features).

Each pair vector is then processed independently by a shared encoder φ implemented as a multi-layer perceptron (MLP):


(1) $$h_{ij}=\varphi (x_{ij}),\;h_{ij}\in R^{H}.$$

Given the set of encoded pair features ${\left\{h_{ij}\right\}}_{n=1}^{N}$ for a decoy, we use a statistical concatenation strategy with a global attention pooling head to aggregate the information. First, we compute standard statistics:


(2) $$h_{\text{sum}}=\sum_{n=1}^{N}h_{n},\;h_{\text{mean}}=\frac{1}{N}\sum_{n=1}^{N}h_{n},\;h_{\text{max}}={\text{max}}_{n}h_{n},$$

(where max is taken element-wise). To additionally capture sparse, high-importance residue pairs, we compute an attention-pooled summary.

We then form the globally pooled representation by concatenating attention pooling with the standard statistics, and append an explicit set-size feature:


(3) $$agg_{k}=\left[h_{\text{attn}}\;\|\;h_{\text{sum}}\;\|\;h_{\text{mean}}\;\|\;h_{\text{max}}\;\|\;\sqrt{N}\right].$$

Where *N* is the number of interface residue pairs of a single decoy.

We then enforce the model to *explicitly represent intra-complex differences* using the information from the *nested complex batch*. For each element *b*, the aggregator normalizes the output using the mean and standard deviation across all *K* decoys for that specific complex in the nested batch.


$$a\hat{g}g_{k}=\frac{agg_{k}-\mu_{K}}{\sigma_{K}+\epsilon }$$

Where $\mu_{K}$ and $\sigma_{K}$ are the mean and standard deviation of *agg* across the *K* decoys for complex *b*

This strategy removes identity bias for the specific complex, i.e. predictions within the same complex can share similar interfaces. By normalizing over the *K* decoys, you remove the “Complex Identity” and force the model to focus on the specific improvements of the decoy in a batch relative to the other decoys of the same complex. Each decoy’s final score is mathematically “aware” of its *K* peers.

Finally, $a\hat{g}g_{k}$ is normalized and passed to a decoder network ρ to predict a scalar score in logit space:


(4) $$\hat{y}=\rho \left(a\hat{g}g_{k}\right)\in R.$$

### 2.4 Composite loss function

To optimize both (i) accurate quality prediction and (ii) consistent ranking of decoys *within each complex*, we minimize a composite objective:


(5) $$L_{\text{total}}=L_{\text{reg}}+\lambda_{\text{rank}}L_{\text{rank}}+\lambda_{\text{dist}}L_{\text{dist}},$$

where $\lambda_{\text{rank}}$ and $\lambda_{\text{dist}}$ are fixed hyperparameters.

### 2.5 Regression loss (L reg)

Let $y_{b,k}\in R$ denote the ground truth DockQ between the antibody and the antigen target in logit space for decoy *k* of complex *b*, and let ${\hat{y}}_{b,k}$ be the model output. We use a Smooth-$\ell_{1}$ (Huber) loss in logit space:


(6) $$L_{\text{reg}}=\frac{1}{BK}\sum_{b=1}^{B}\sum_{k=1}^{K}{\text{Huber}}_{\beta }\left({\hat{y}}_{b,k}-y_{b,k}\right),$$

with robustness parameter β.

#### 2.5.1 Soft spearman ranking ($L_{\text{rank}}$)

To directly optimize the ordering of decoys within each complex, we use a differentiable Spearman’s rank correlation loss. We first map logit outputs to DockQ space:


(7) $$p_{b,k}=\sigma ({\hat{y}}_{b,k}),\;t_{b,k}=\sigma (y_{b,k}),$$

where σ(·) denotes the sigmoid.

For a score vector $s\in R^{K}$, we approximate the rank of element *k* via pairwise sigmoid comparisons with temperature τ:


(8) $${\hat{r}}_{i}(s)=1+\sum_{j\neq i}\sigma \left(\frac{s_{i}-s_{j}}{\tau }\right).$$

Let $\hat{r}(p_{b})$ and $\hat{r}(t_{b})$ denote the soft-rank vectors for predictions and targets of complex *b*. We then compute a Spearman-style correlation by penalizing squared rank discrepancies:


(9) $$\rho_{\text{soft}}(b)=1-\frac{6\sum_{k=1}^{K}{\left({\hat{r}}_{i}(p_{b})-{\hat{r}}_{i}(t_{b})\right)}^{2}}{K(K^{2}-1)}.$$

The ranking loss is defined as


(10) $$L_{\text{rank}}=\frac{1}{B}\sum_{b=1}^{B}\left(1-\rho_{\text{soft}}(b)\right).$$

Since ranking is only meaningful when the *K* decoys for a complex exhibit non-trivial variation in quality (i.e. they do not fall strictly in the same structural cluster), we modulate the per-complex ranking loss by a continuous relevance weight based on the standard deviation of target scores:


(11) $$w_{b}=\text{clip}\left(\frac{\text{Std}(t_{b})}{\gamma },0,1\right),$$

and use the gated loss


(12) $$L_{\text{rank}}=\frac{1}{B}\sum_{b=1}^{B}w_{b}\left(1-\rho_{\text{soft}}(b)\right),$$

where γ is a scaling constant.

##### 2.5.1.1 *Distance preservation* ($L_{\mathrm{dist}}$*)*

While $L_{\text{rank}}$ enforces correct ordering, it does not constrain the *magnitude* of predicted quality gaps. We therefore include a distance preservation objective operating on DockQ probabilities. For each complex *b*, we match pairwise absolute score differences between predictions and targets:


(13) $$L_{\text{dist}}=\frac{1}{B}\sum_{b=1}^{B}\sum_{1\leq k<j\leq K}{\left(\left|p_{b,k}-p_{b,j}\right|-\left|t_{b,k}-t_{b,j}\right|\right)}^{2}.$$

This term encourages the predicted score to respect not only the rank order but also the relative separation of decoys in quality space.

## 3 Results and discussion

### 3.1 Evaluation metrics

The performance of an antibody–antigen scoring model is evaluated based on two criteria: (i) its ability to rank the full ensemble in a way, i.e. consistent with structural quality and (ii) its ability to retrieve the top*K* highest-quality decoys from an ensemble of predicted structures.

All results are reported on the hold-out test split, and for each complex, we restrict evaluation to a *strict intersection* of decoys for which *all* methods provide valid predictions. All metrics below are computed on this matched subset. This subset comprises 1009 complexes and 50433 samples.

For a given complex, let $t^{⋆}={\text{max}}_{i}\;t_{i}$ denote the best achievable quality (DockQ) within the decoys pool. Each method assigns a score ${\hat{s}}_{i}$ and thus induces a ranking. We denote by $\text{Top}K(\hat{s})$ the indices of the *K* highest-ranked decoys, and define the best recovered quality within the shortlist as


(14) $$t_{\text{max}}^{\left(K\right)}=\underset{i\in \text{Top}K(\hat{s})}{\max}t_{i}.$$

Here, we report:

- *Tiered Top-K Success (threshold-based).* A method succeeds if the shortlist contains at least one decoy above a quality threshold τ:
  (15) $$\text{Success}(K;\tau )\;\;\Leftrightarrow \;\;t_{\text{max}}^{\left(K\right)}\geq \tau ,\;\tau \in \{0.23,0.50,0.80\}.$$
  These thresholds correspond to acceptable, medium, and high-quality regimes of the DockQ metric as defined in (Basu and Wallner 2016).
- *Real Success (best-decoy retrieval).* A stricter criterion that succeeds only if the method retrieves the *single* best decoy in the pool within the top-*K*:
  (16) $$\text{RealSuccess}(K)\;\;\Leftrightarrow \;\;\text{arg}\underset{i}{\max}t_{i}\in \text{Top}K(\hat{s}).$$
- *Relaxed Near-Optimal Success (gap-based).* To capture the *usage perspective*, we also credit near-optimal retrieval when the selected decoy is equivalent to the best available structure. We quantify the quality gap as:
  (17) $$\begin{array}{c} \Delta_{K}=t^{⋆}-t_{\text{max}}^{\left(K\right)},\\ {\text{Success}}_{\text{relaxed}}(K;\delta )\Leftrightarrow \Delta_{K}\leq 0.05 \end{array}$$

This metric distinguishes failure to retrieve the best model from cases in which the returned model lies within a small DockQ tolerance and is therefore equally valuable from a practical perspective. This metric is shown in Table 2 under the Real Success Rate (both for the best and the relaxed version).

**Table 2:** Performance of ABAG-Rank and baselines at selecting the best-interface quality Ab–Ag model among the top-*K* ranked samples.

Method	**Top-*K* success rate (%) (** ↑ **)**				**Real success rate (%) (** ↑ **)**					
	**Acceptable**		**Medium**		**Best decoy**		**Relaxed decoy**		**Relaxed decoy**	
	**(** τ = 0.23 **)**		**(** τ = 0.50 **)**		(Strict)		**(** Δ≤0.01 **)**		**(** Δ≤0.05 **)**	
	**T-1**	**T-5**	**T-1**	**T-5**	**T-1**	**T-5**	**T-1**	**T-5**	**T-1**	**T-5**
Oracle (Upper Bound)	58.77	58.77	44.80	44.80	100.00	100.00	100.00	100.00	100.00	100.00
Random	36.97	46.18	31.02	38.26	9.42	23.98	13.58	29.04	45.09	66.90
AlphaFold3	43.01	46.18	**36.77**	39.15	10.21	25.57	14.87	30.23	51.14	64.92
DeepRank-Ab	42.62	48.07	36.27	40.24	9.61^ns^	25.87	14.57	30.92	51.54	69.08
ipSAE	40.83	46.48	35.68	38.55	8.82^ns^	25.57	13.58	30.72	49.75	65.81
pDockQ	41.53	47.08	35.88	39.94	8.92^ns^	26.36	13.68	31.52	49.16	67.00
pDockQ2	42.72	46.78	36.08	38.85	10.70^ns^	24.98	15.06	29.34	50.74	67.00
ABAG-Rank	**44.90**	**49.26**	36.47	**40.34**	13.08*	**30.23**	**15.76**	**34.39**	**54.11**	**70.56**

τ represents the true DockQ threshold. Best performance is indicated in bold. Statistical significance against AlphaFold is denoted by ^ns^ (not significant),

* (*P* < .05)

#### 3.1.1 Ranking correlation metrics

To evaluate the quality of the full ordering (beyond shortlist retrieval), we report:

- *Per-complex Spearman correlation.* For each complex *c*, we compute $\rho_{c}=\text{Spearman}(\{{\hat{s}}_{i}\},\{t_{i}\})$, and summarize performance across complexes. This measures whether each method preserves relative order within an ensemble. While this is a standard metric, the per-complex Spearman correlation is especially relevant for complexes where the distribution of interface quality exhibits greater variance, and less relevant for complexes that cluster in a similar-quality region (i.e., for a complex where all decoys fall in a cluster of very similar DockQ, the absolute correct ordering of the decoys is often irrelevant, while correct ordering for complexes where the variance of DockQ value of the decoys is much more important).
- *Global Spearman correlation.* We additionally compute the Global Spearman correlation after concatenating all matched decoys across complexes. This reflects overall monotonic agreement and is sensitive to cross-complex calibration.

The performance of the ABAG-Rank model referred to throughout the main text includes the full set of input features as defined in the Methods.

### 3.2 ABAG-Rank achieves the best performance at ranking interface quality

**Figure 3:**
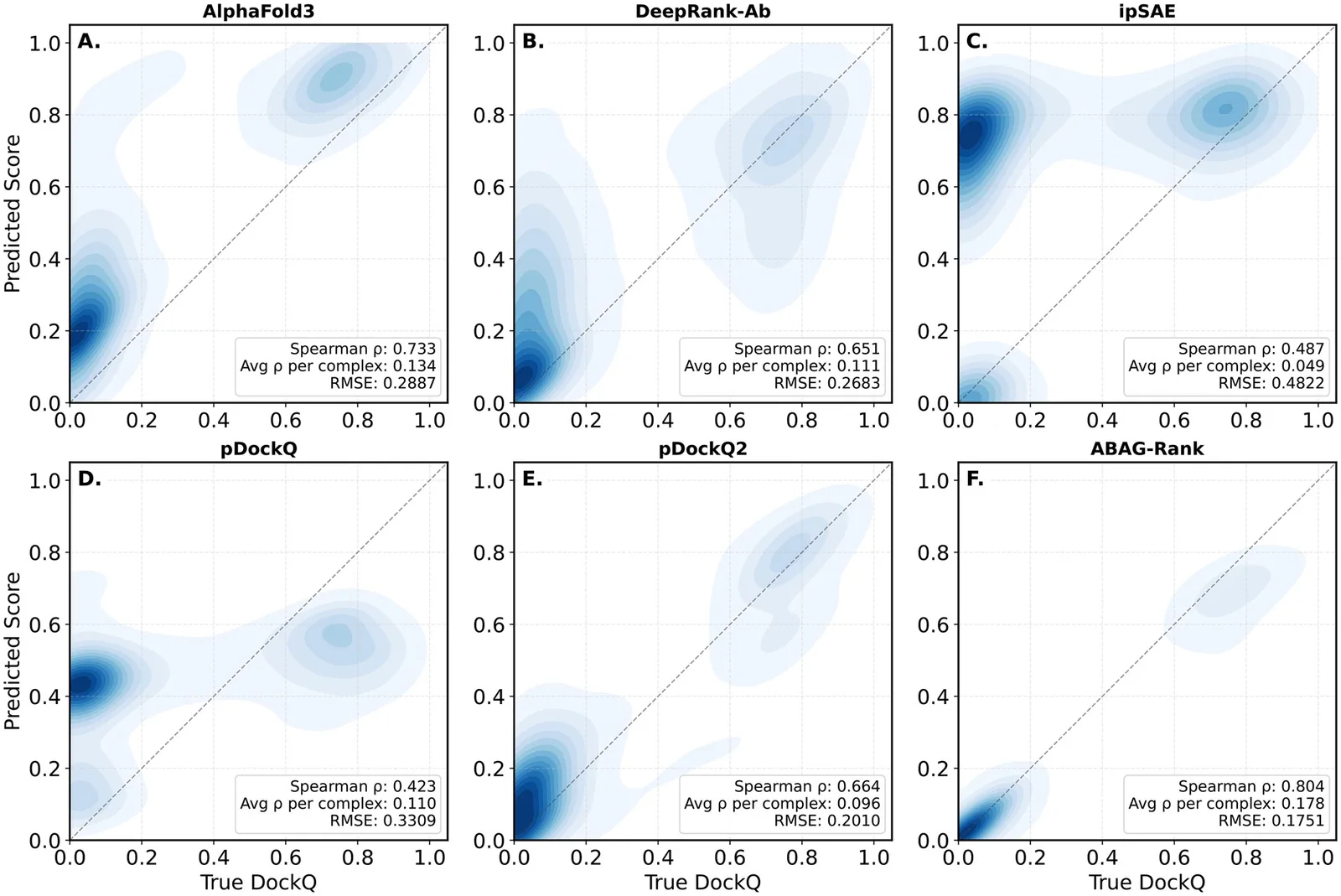
Distribution of predicted scores as a function of the ground-truth DockQ score for (A) AlphaFold3 ranking confidence, (B) DeepRank-Ab, (C) ipSAE, (D) pDockQ, (E) pDockQ2 and (F) ABAG-Rank.

We first evaluate ABAG-Rank and the baseline methods on their ability to rank interface model quality at both global and per-complex levels. Surprisingly, all baselines, in general, perform either worse than or, on a subset of metrics, on par with AF3’s ranking confidence, and thus underperform ABAG-Rank. ABAG-Rank outperforms both AF3 ranking confidence and DeepRank-Ab in global and per-complex Spearman correlation between predicted scores and ground-truth DockQ values, while also predicting the interface quality with the lower numerical error (Table 1). Although the overall prediction quality is better for complexes similar to the AF3 training set fromm2025, we do not observe any difference in the ability to rank complexes by similarity to the AF3 training set; see Table S2. Crucially, while being multiple orders of magnitude faster than DeepRank-Ab, ABAG-Rank shows a statistically significant improvement over AF3 confidence in per-complex Spearman correlation, whereby DeepRank-Ab does not. AF3 and DeepRank-Ab tend to substantially overestimate the interface quality of models with incorrectly predicted binding pose, assigning high predicted scores when the ground-truth DockQ score is very low (Fig. 3). In contrast, ABAG-Rank is much less prone to this failure mode and scores the interface quality more robustly, thereby reducing the number of high-scoring false positives. ABAG-Rank also achieves the highest AUC across classification thresholds defined by DockQ cutoffs (Acceptable >0.23, Medium >0.50, and High >0.80), indicating stronger discrimination between low- and high-quality binding modes (Table 1). When comparing the predicted ranking scores to the underlying ground-truth DockQ distribution, we observe that all methods except ABAG-Rank fail to reproduce the shape of the true interface quality distribution (Fig. S1). In contrast, ABAG-Rank most closely matches the ground-truth DockQ distribution among the evaluated methods. Notably, the internal AF3 confidence metrics pTM and ipTM exhibit a high-density concentration around very high scores, which might partly explain their tendency to assign overly confident scores even to low-quality models. Among the two, ipTM nevertheless more closely follows the overall shape of the ground-truth DockQ distribution; see Figs. S2 and S3.

**Table 1:** Performance of ABAG-Rank and baselines at ranking Ab–Ag models according to their interface quality.

Method	**Global Regression Metrics**			**AUC Classification (** ↑ **)**			Inference
	**RMSE (** ↓ **)**	**Spearman (** ρ **) (** ↑ **)**		**Threshold (DockQ)**			**Time (s) (** ↓ **)**
		Global	Per Cplx	**Acc (** >0.23 **)**	**Med (** >0.50 **)**	**High (** >0.80 **)**	per Sample
Random	0.489	−0.000	0.004	0.500	0.502	0.495	–
AlphaFold3	0.289	0.733	0.134	0.920	0.924	0.870	–
DeepRank-Ab	0.268	0.651	0.111**	0.876	0.885	0.883	23.81^†^
ipSAE	0.482	0.487	0.049***	0.759	0.761	0.711	3.44
pDockQ	0.331	0.423	0.110*	0.758	0.780	0.692	1.95
pDockQ2	0.201	0.664	0.096***	0.890	0.918	**0.915**	–
ABAG-Rank	**0.175**	**0.804**	0.178**	**0.935**	**0.946**	0.910	≤ 0.05*

Best performance is indicated as bold. RMSE: Root Mean Square Error, AUC: Area Under the Curve. Arrows down or up indicate the direction of preference. Statistical significance is denoted by

*** (*P* < .001).

**(*P* < .01)

*(*P* < .05)

† for DeepRank-Ab denotes default inference settings of the published model.

* for ABAG-Rank reports the time including the dataset preparation and the full inference pipeline with a batch size of 10.

### 3.3 ABAG-Rank improves retrieval of the best-available interface models

In practice, the most relevant objective is to retrieve the best available model of an Ab–Ag complex given a set of structural decoys, since ranking the predicted models alone does not necessarily lead to identification of the correct binding mode. We therefore evaluate whether ABAG-Rank can correctly identify the DockQ quality class of the top-*K* ranked samples and retrieve the sample with the highest possible interface quality within the top-*K* set. ABAG-Rank demonstrates either improved or on-par performance compared with AF3 ranking confidence and DeepRank-Ab in identifying *Acceptable* and *Medium* quality interfaces among the top-1 and top-5 ranked samples (Table 2). Although the overall prediction quality is better for complexes similar to the AF3 training set fromm2025, ABAG-Rank performance is unaffected by test-to-train sequence interface redundancy (Table S1, however, when analysing the structural similarity, it’s performances drop slightly for global metrics, while still remaining state of the art, and a bigger drop in performance in per complexes Spearman correlation, suggesting that the per complex spearman correlation could be strongly associated by structures seen in the training set and limited generalizability in new unseen structures S2).

When the task is defined more stringently as retrieving the best overall available interface-quality sample (Real Success), ABAG-Rank achieves SOTA, although the success rate remains quite low. Conceptually, retrieval of the *Best Decoy* is inherently challenging because it requires identifying the single numerically highest-DockQ sample, even when several alternative predictions could possess nearly identical interface and, consequently, numerically similar DockQ scores. Interestingly, when the criterion is therefore relaxed to allow a DockQ deviation of at most 0.05 from the best possible solution, the problem becomes substantially more tractable, indeed reflecting the presence of many near-optimal samples clustered close to the top-scoring decoy. Under this relaxed definition, ABAG-Rank again achieves the best performance among all evaluated methods (Table 2).

This trend is further illustrated in Fig. S4. In the top-*K* retrieval setting, all methods progressively approach the Oracle upper bound as *K* increases. In contrast, when considering only the single top-ranked prediction as a function of the sample pool size, all methods exhibit clear performance saturation and consistently remain below the best available sample. This behaviour is consistent with the previous observation that exact best-decoy retrieval is numerically stringent. Even as more samples become available, the ranking methods, including ABAG-Rank, struggle to reliably assign the optimal solution to the top-ranked decoy. This can be seen in a more pronounced way in a subset test set where we sampled 500 decoys per complex, see Figs. S3–S6.

### 3.4 Ablation and generalizability study

To assess the contribution of individual input features to ABAG-Rank performance, we conducted an ablation study in which separate models were trained with specific input features removed (S7). We report ablation on the following variants: retraining with unfiltered dataset, removal of ESM features, without iptm and ptm, without ESM and PAE and without ESM, PAE and iptm and ptm. For the overall best interface-quality model selection, we find that the model that uses only interface spatial $C_{\alpha }$ distances and one-hot residue identity encoding (i.e. without ESM, PAE and (i)pTM), already improves over both AF3 ranking confidence and DeepRank-Ab in the global metrics, by reducing the number of high-scoring false positives (Fig. S8). This suggests that simple structural and sequence-level descriptors might already be sufficient to provide a good initial estimate of Ab–Ag interface quality. Notably, DeepRank-Ab, despite relying on physics-derived features computed with HADDOCK, produces even a higher number of highly-scored incorrect predictions and underperforms the AF3 ranking confidence. The addition of (i)pTM and PAE to ABAG-Rank progressively improves its performance, while the inclusion of ESM embeddings yields the best overall performance (Tables S5 and S6). This suggests that the predictive information of AF-like models provides strong features that, with the help of additional information and recalibration, helps to provide a more reliable interface quality estimate.

### 3.5 Generalizability of ABAG-Rank to Boltz2

To study the generalizability of ABAG-Rank to another model we generated the test set using Boltz2 instead of AF3 using the same method for AF3. Since Boltz2 differs from AF3 in architecture and training procedure ABAG-Rank trained on AF3 values generalizes well to Boltz2 features on global metrics, but its performance decreases in the per complex spearman, due to different feature distributions Fig. S10, a mode noticed also when analysing generalizability of per-complex Spearman ρ on unseen structure in the structural similarity analysis mentioned before. Performing a fine-tuning on a small subset of the training set improves RMSE and global correlation S7, decreasing the amount of false positives for incorrect samples Fig. S9. This limited fine-tuning further improved performance on the global regression and AUC classification metrics (Table S7), demonstrating that ABAG-Rank can be recalibrated to decoys generated by a different folding architecture using a relatively small method-specific adaptation set. Despite this approach providing more aligned results to the ground truth, it does not achieve state-of-the-art performance on the top-*K* and native success rate, and a more extensive retraining of the model is necessary. For the fine-tuning, we selected 409 complexes, resampled with Boltz2, representing around 10% of the original training set, for a total of 7670 samples. We ran fine-tuning of the new dataset on a single GPU (1× A100-40GB) for around 6 h with a learning rate of 0.0001 (ten times smaller than the original training). Results are reported in Tables S7 and S8 and Figs. S9–S12.

### 3.6 Performance analysis by complexity factor

To better understand the performance and failure cases of ABAG-Rank, we analyse the SAbDab test set across three factors: (i) *oracle quality*, whether the true best decoy is near-native; (ii) *landscape flatness*, whether the DockQ scores within a complex are spread enough for ranking to be meaningful; and (iii) *structural properties*, including interface size and antibody format. Together, these factors explain most cases where ABAG-Rank does not retrieve the best decoy, and show that many failures are tied to the input data rather than the model. Figure S13 shows how decoy-set difficulty (DockQ spread quartiles) affects both retrieval and ranking quality. Figure S15 examines the effect of interface size on performance. Figure S17 compares VHH/single-domain and IgG Fab antibody formats. Finally, Fig. S16 provides a full taxonomy of failure conditions on the test set.

## 4 Conclusions

This work introduces ABAG-Rank, a deep neural network for ranking the interface quality of antibody–antigen models. ABAG-Rank achieves state-of-the-art performance on this task and outperforms existing baselines at both the global and per-complex levels, while also more effectively retrieving the best available structural solutions from AF3-generated ensembles. Importantly, the model addresses one of the key limitations of AF3 by substantially reducing the number of high-scoring false positives and assigning lower confidence to incorrect binding modes. Our results further show that the combination of AF3 internal confidence metrics, protein language model embeddings and simple geometric interface descriptors provides a strong estimate of interface quality without requiring expensive physics-based calculations. In addition, ABAG-Rank is multiple orders of magnitude faster than another deep learning baseline, DeepRank-Ab, making it well suited for large-scale evaluation of AF3 predictions in exploratory Ab–Ag docking studies. At the same time, the absolute performance of ABAG-Rank remains bounded by the generative capacity of AF3: if the sampled decoy pool does not contain high-quality conformations, no ranking model can recover the correct solution. Thus, while ABAG-Rank provides an effective solution to the ranking bottleneck, improving the generation of accurate candidate structures remains a fundamental challenge for the field.

## Supplementary Material

btag663_Supplementary_Data
