# Supplementary material for: ABAG-Rank: improving model selection of AlphaFold antibody–antigen complexes by learning to rank
Source: Bioinformatics. 2026 Sep 4;42(9):btag663. doi: 10.1093/bioinformatics/btag663 (PMC13588714; doi:10.1093/bioinformatics/btag663)
Supplement: Supplementary file 1 [file btag663_supplementary_data.pdf]

Supplementary Information

Supplementary Figures

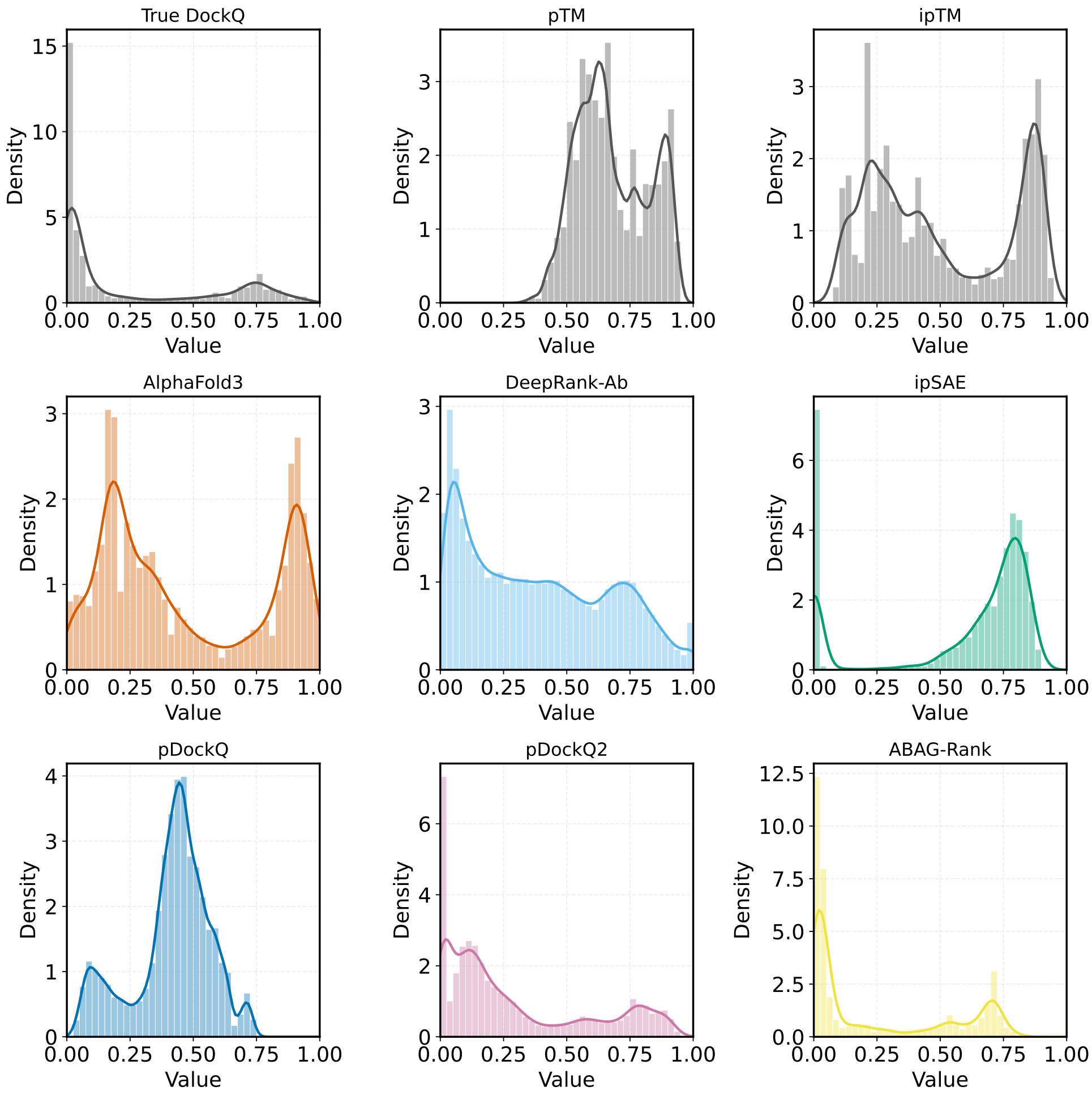

Fig. S1. Distribution of the structural quality of AF3 predictions and the performance of the ranking methods along with pTM and iPTM for 1091 hold-out Ab-Ag complexes.

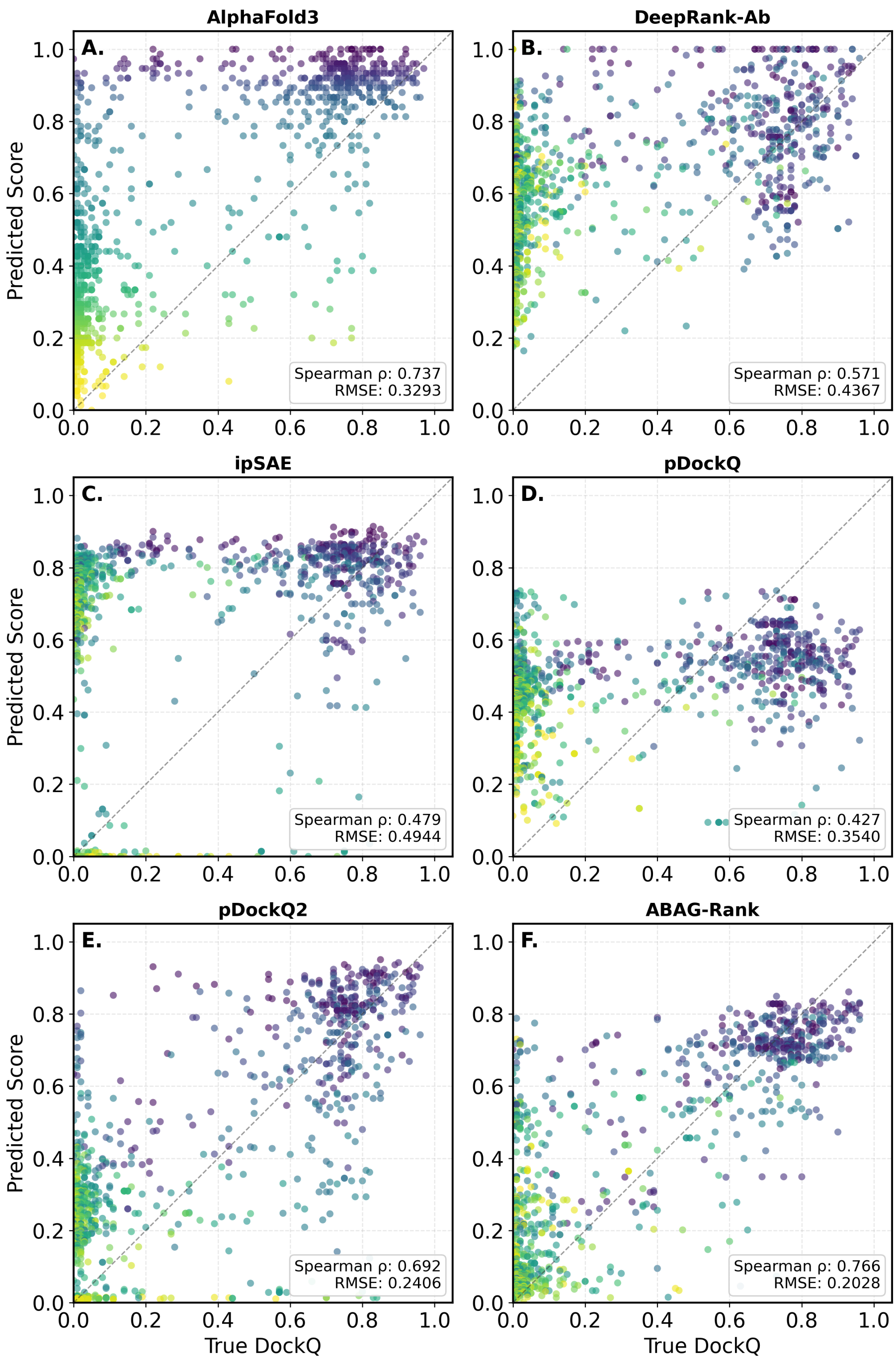

Fig. S2. Top 1 selection baselines scatter plots: Comparison of ABAG-Rank and the baseline performance on the task of selecting the best-quality interface sample (top-1) for 1091 hold-out complexes. (F) Denotes the models reported in the main text, which rely upon ESM, PAE and (i)pTM features.

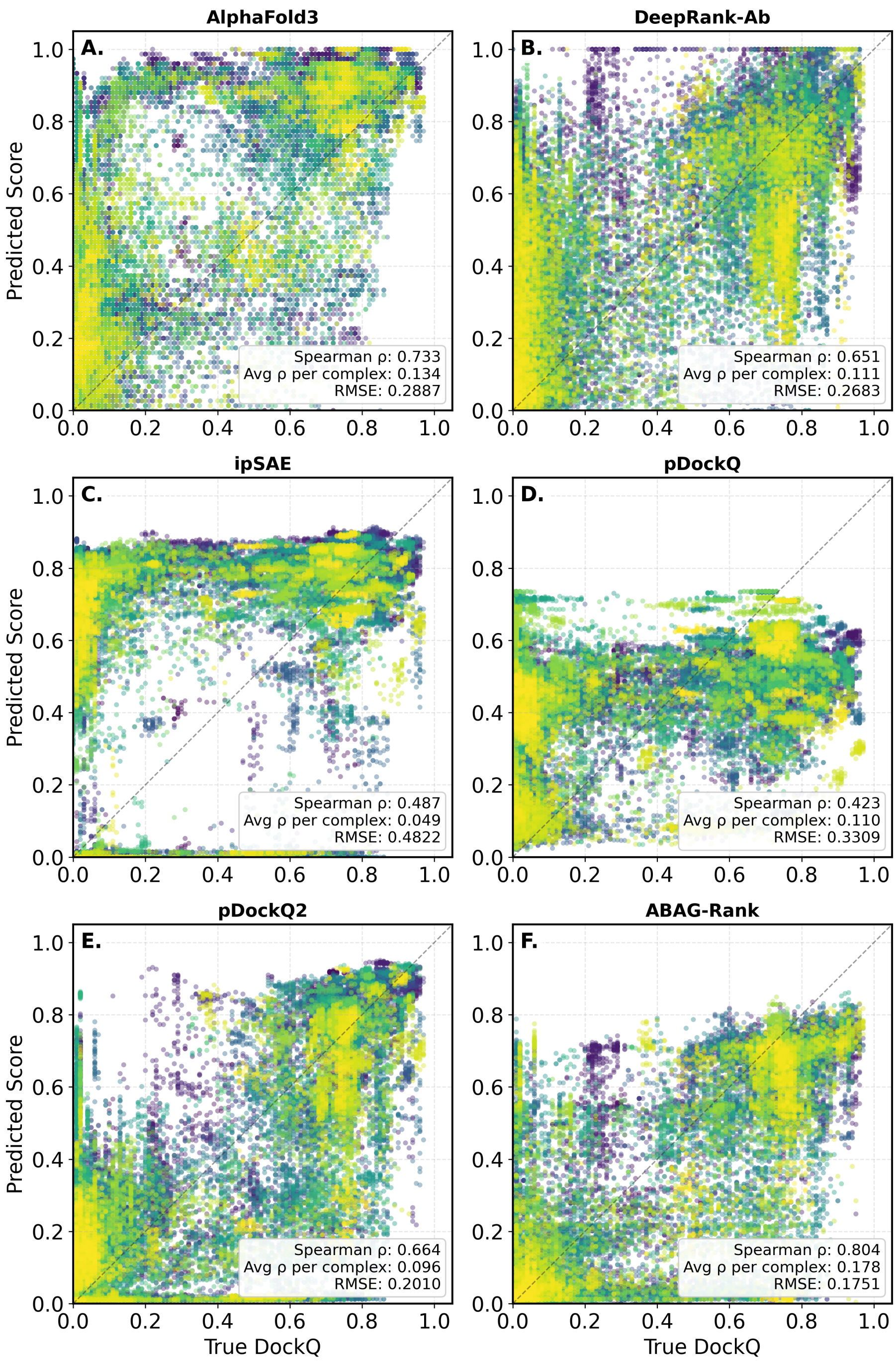

Fig. S3. All samples baselines scatter plots: Comparison of ABAG-Rank and baseline models on global ranking of Ab-Ag models according to their interface quality for all 50433 samples across 1091 hold-out complexes. (F) Denotes the models reported in the main text, which rely upon ESM, PAE and (i)pTM features.

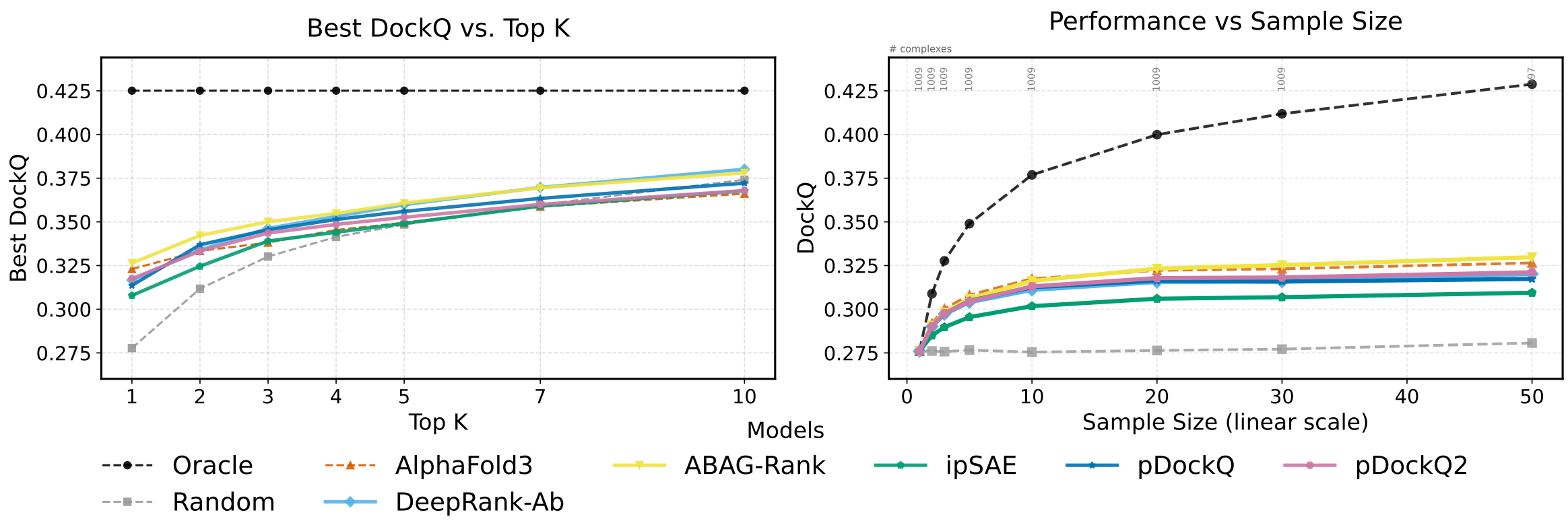

Fig. S4. Left: Retrieval performance of the best-scoring model as a function of top-K models. DockQ is computed across the top-K models on the entire dataset, comprising 1091 hold-out complexes not seen during training. **Right:** Ranking performance as a function of decoy pool size, measured by the mean DockQ versus the sample set size N. The Oracle and Random curves denote the upper bound and the random-ranking baseline, respectively.

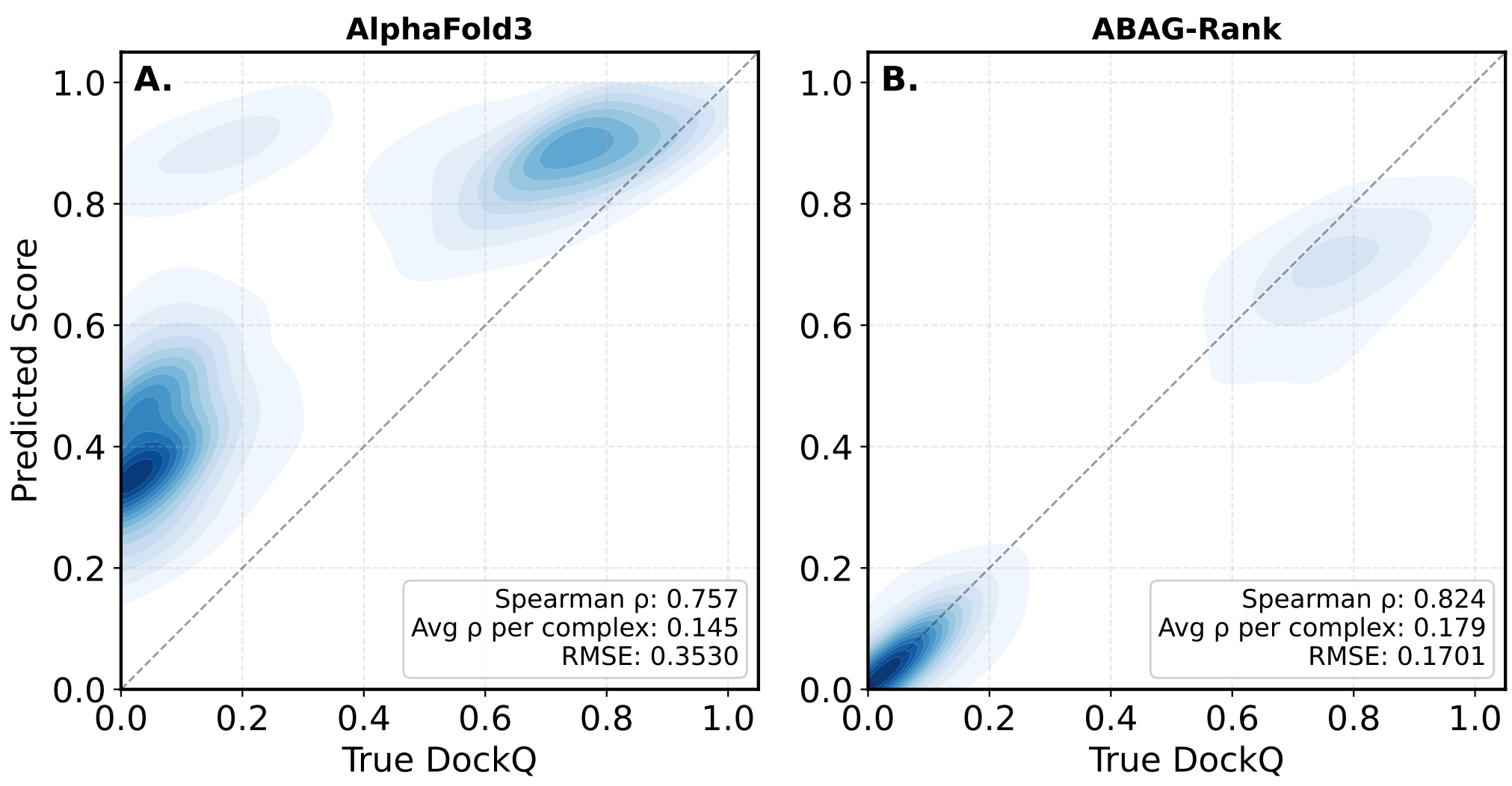

Fig. S5. Density 500 samples: Density plot of a 236-complex subset of the test set sampled for 500 structures for each complex. For a total of 108434 samples

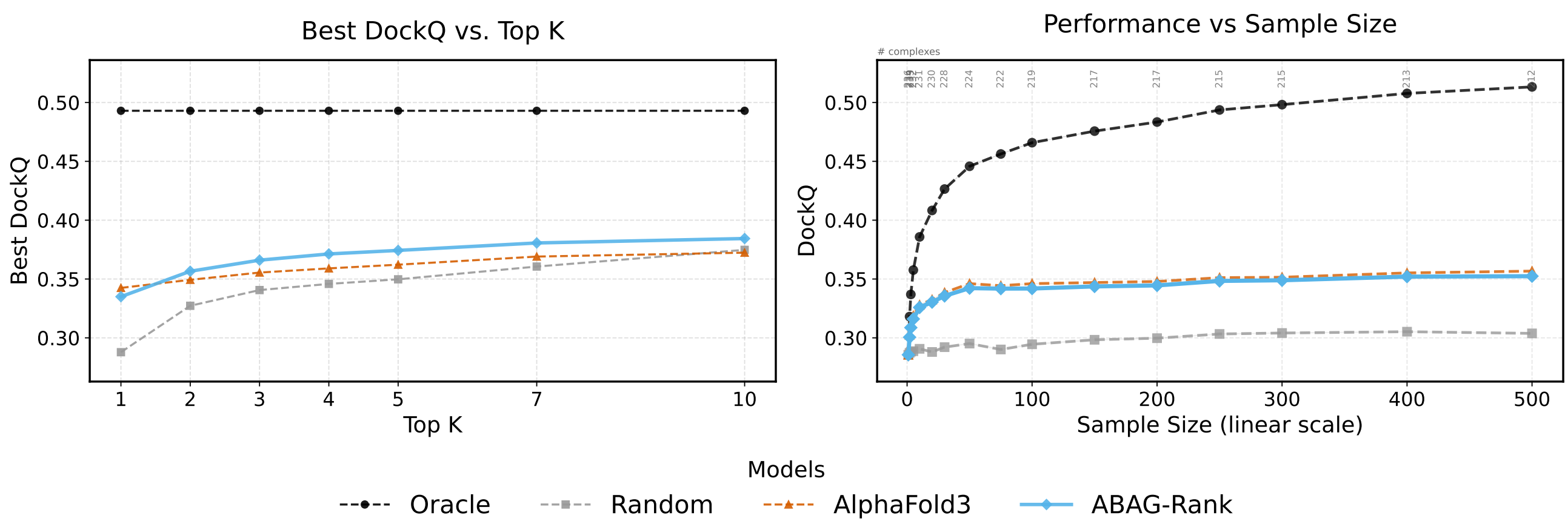

Fig. S6. 500 samples: Left: Retrieval performance of the best-scoring model as a function of top-K models. DockQ is computed across the top-K models on the entire dataset, comprising 1091 hold-out complexes not seen during training. **Right:** Ranking performance as a function of decoy pool size, measured by the mean DockQ versus the sample set size N. The Oracle and Random curves denote the upper bound and the random-ranking baseline, respectively.

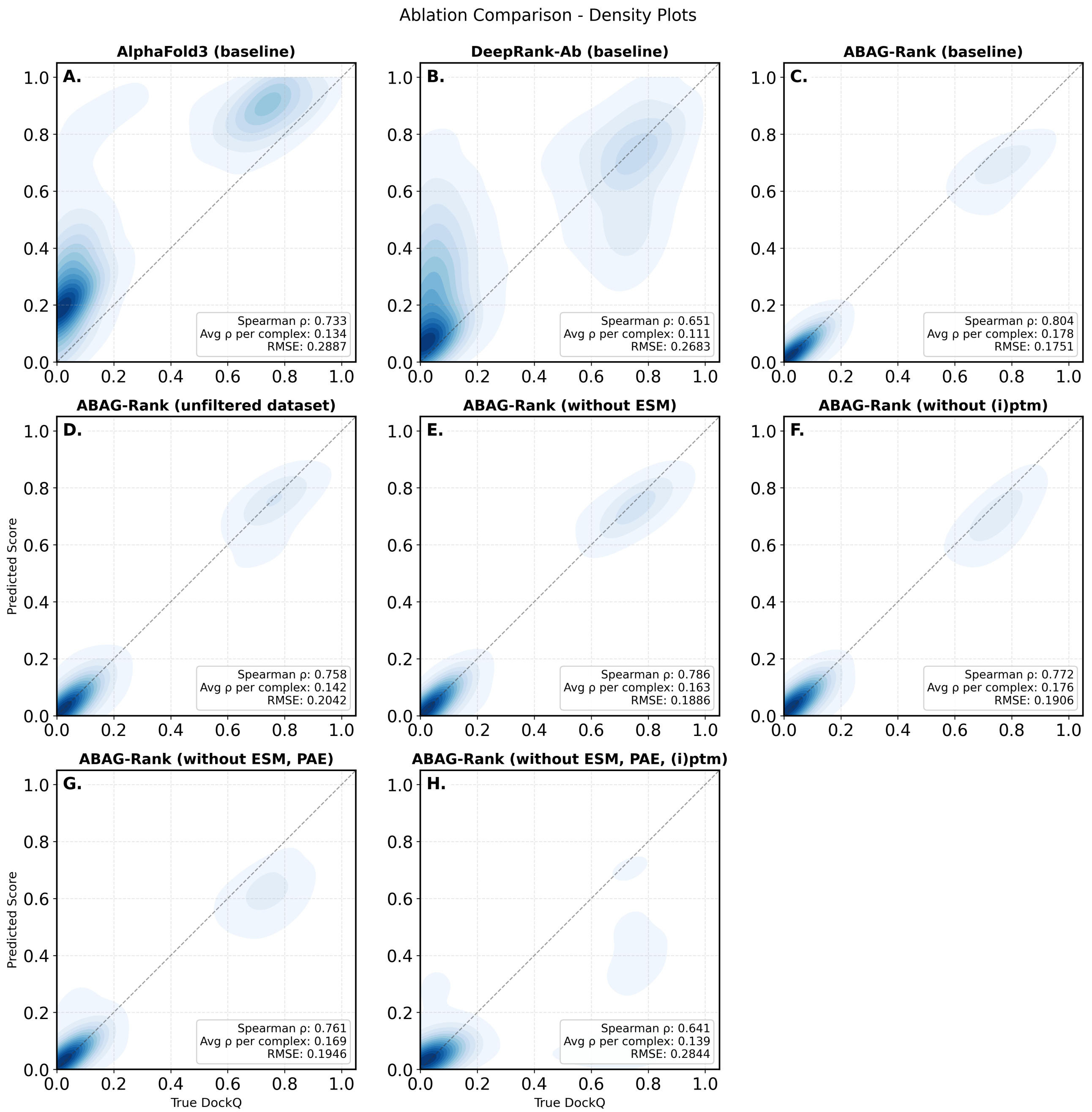

Fig. S7. Density Plot Ablation: Distribution of predicted scores as a function of the ground-truth DockQ score for the ABAG-Rank ablation variants.

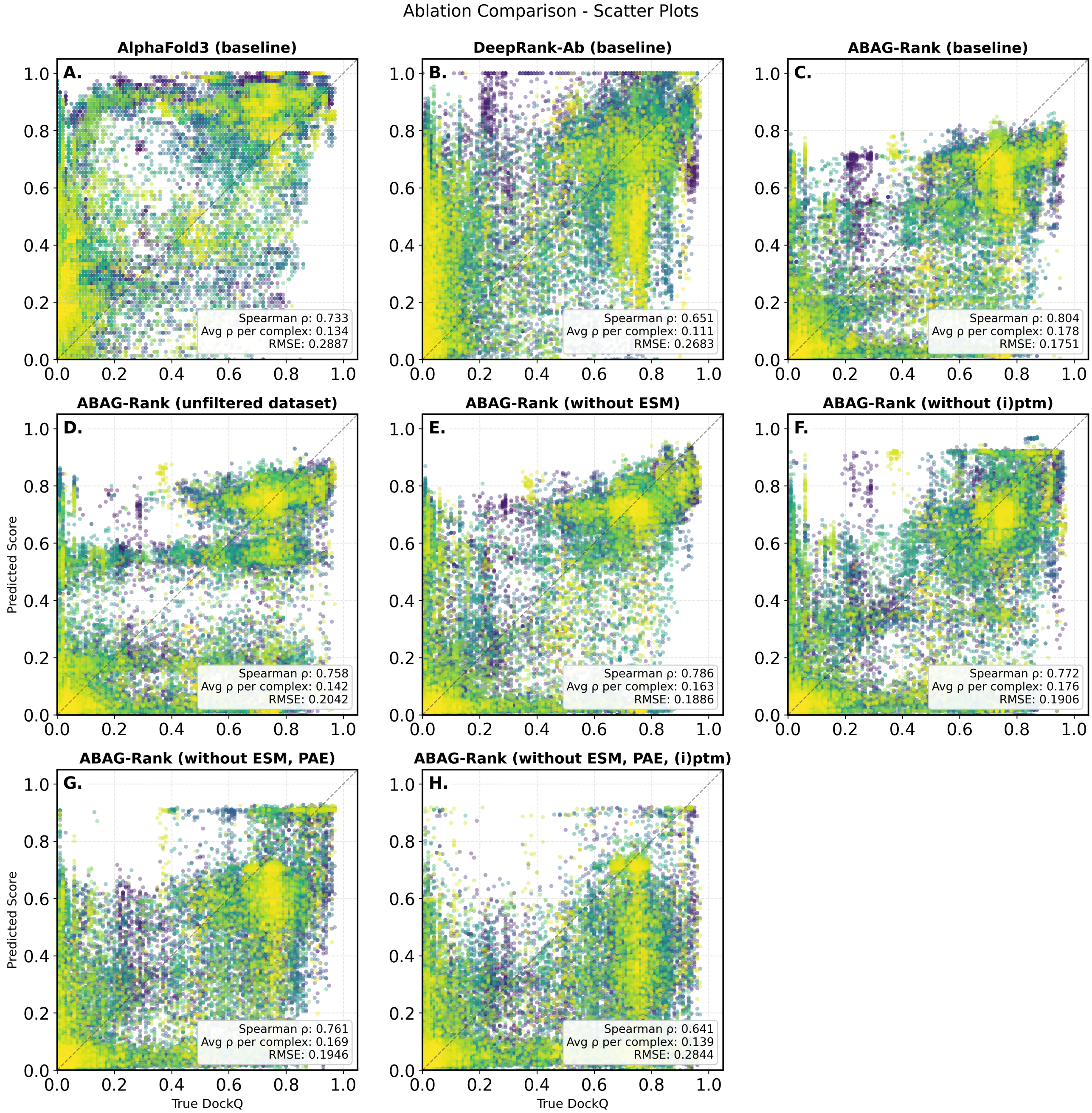

Fig. S8. Scatterplot all test samples ablation: Comparison of ABAG-Rank ablation variants on global ranking of Ab-Ag models according to their interface quality for all 54550 samples across 1091 hold-out complexes.

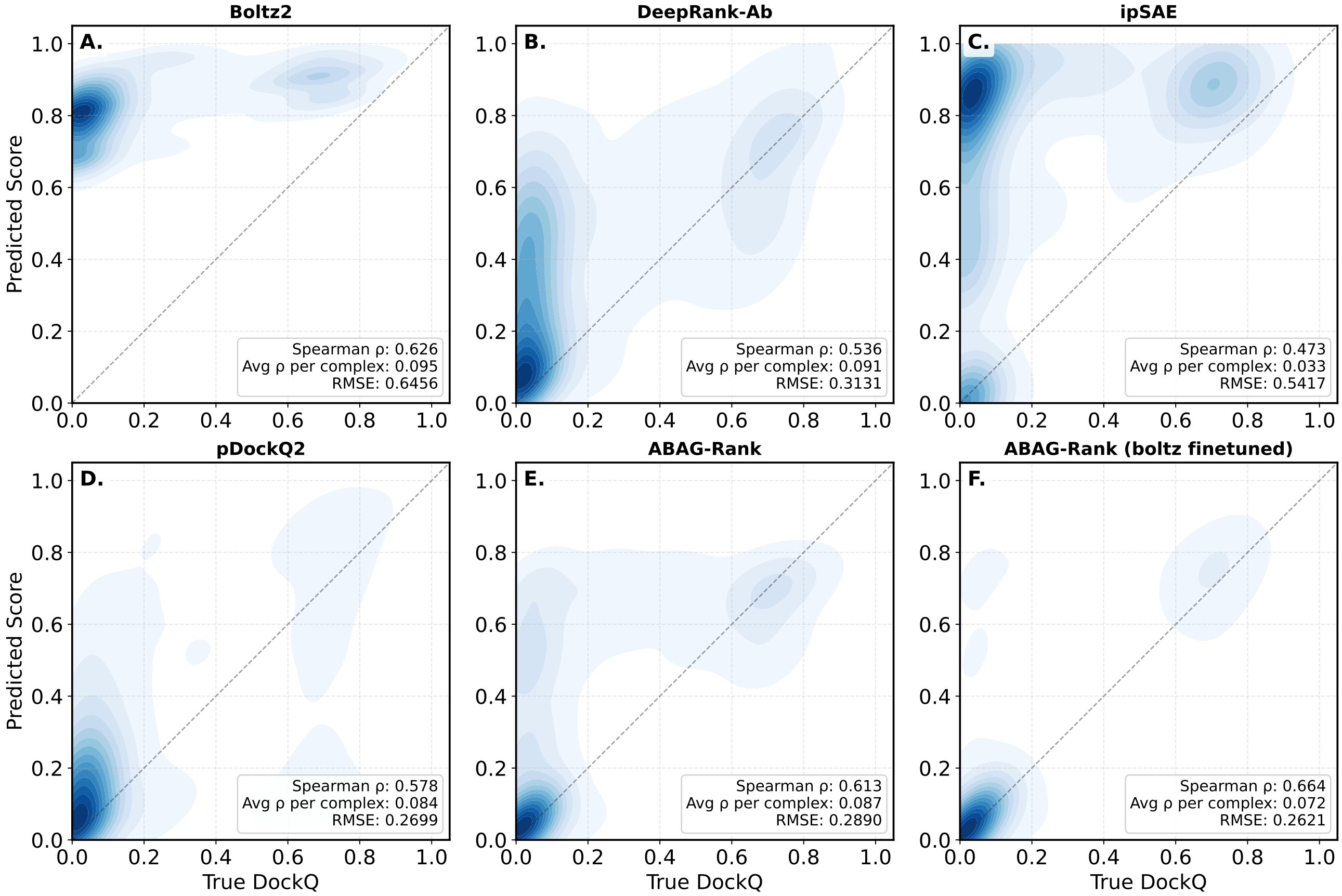

Fig. S9. Boltz2 Density plot: Distribution of predicted scores as a function of the ground-truth DockQ score for Boltz2 predictions.

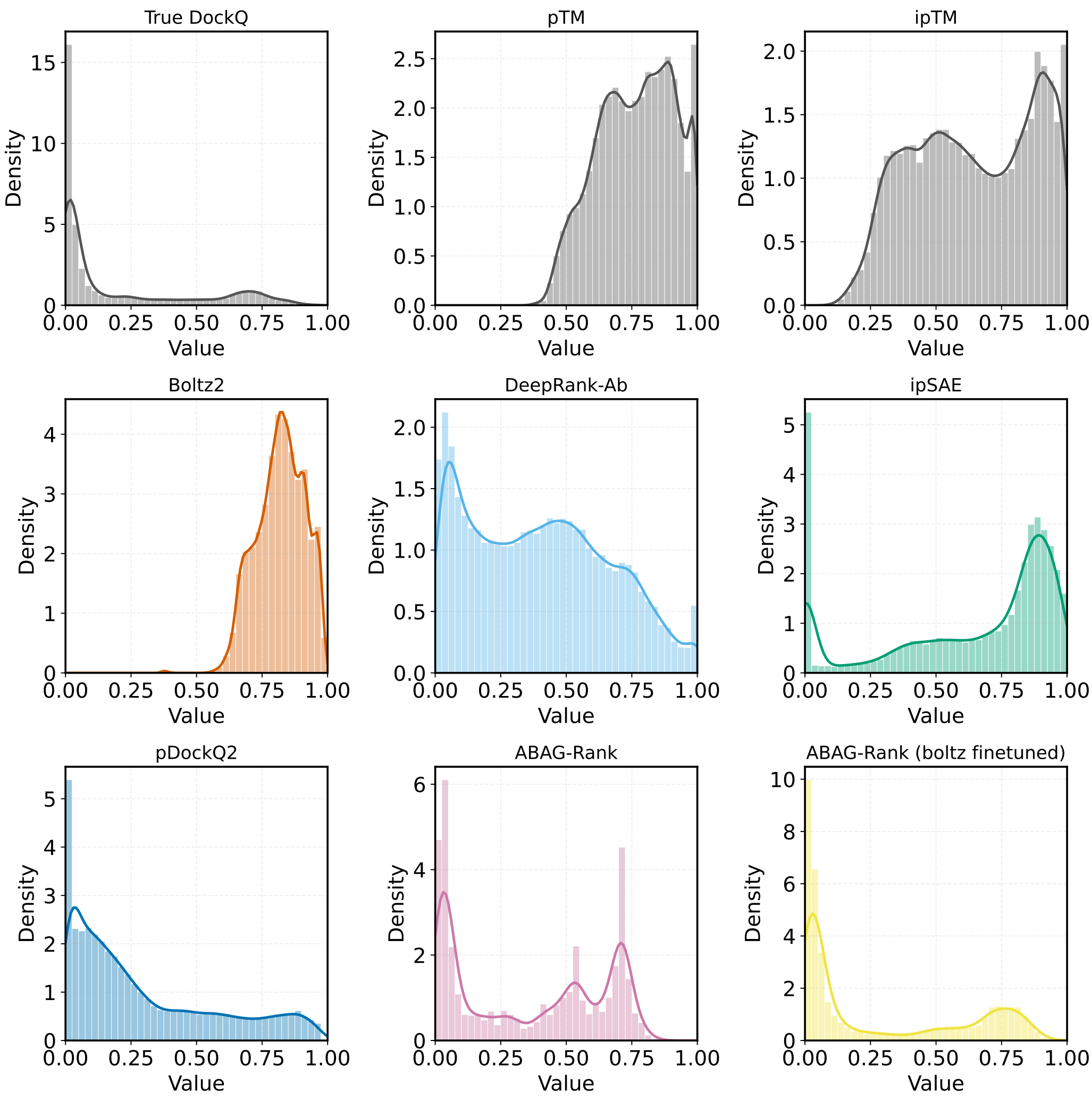

Fig. S10. Boltz2 distribution plot: distribution of the structural quality of Boltz2 predictions and the performance of the ranking methods along with pTM and iPTM for 1091 hold-out Ab-Ag complexes.

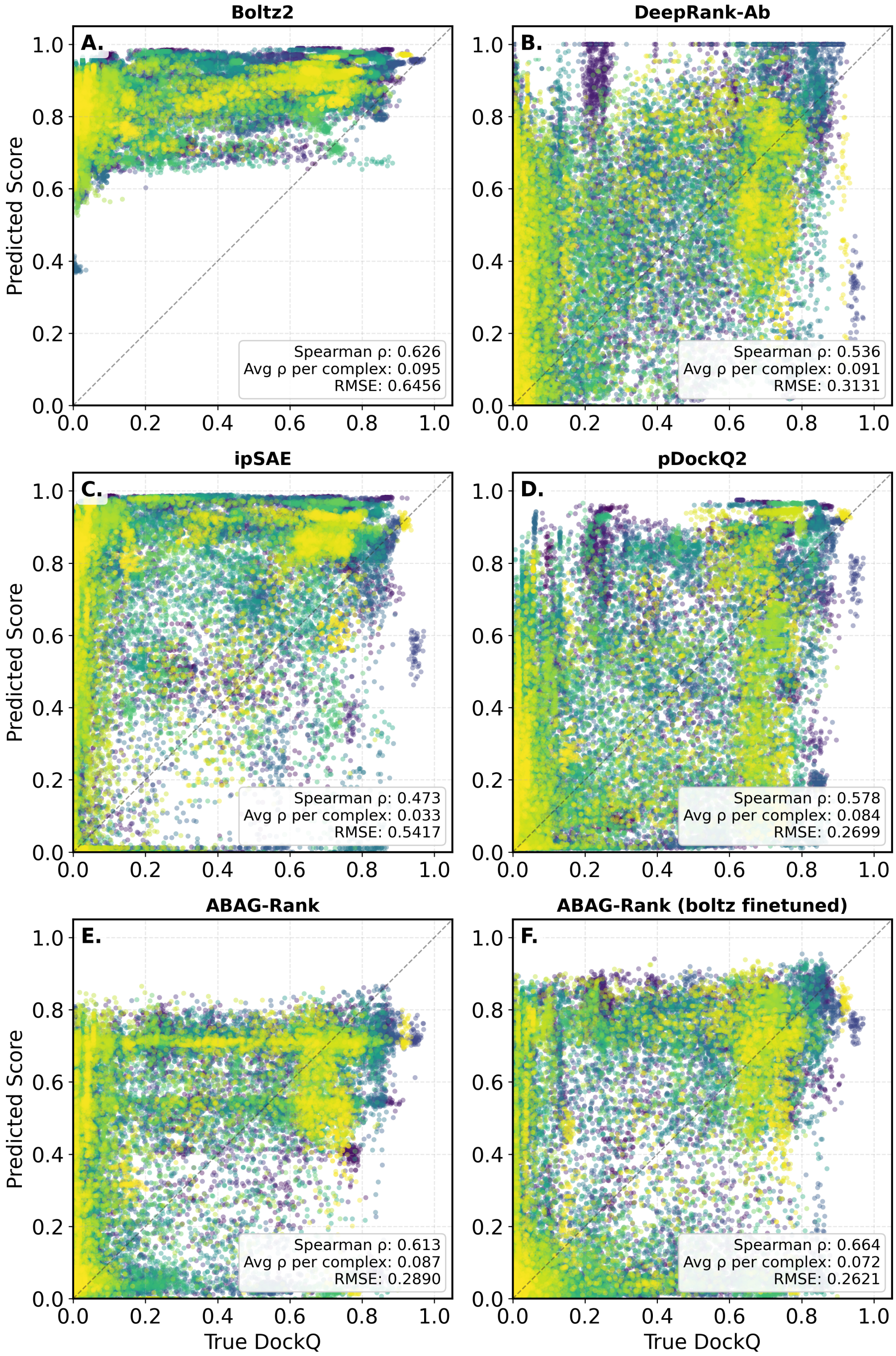

Fig. S11. Boltz2 scatter plot all samples: Comparison of ABAG-Rank and baseline models on global ranking of Ab-Ag models according to their interface quality on Boltz2 predictions

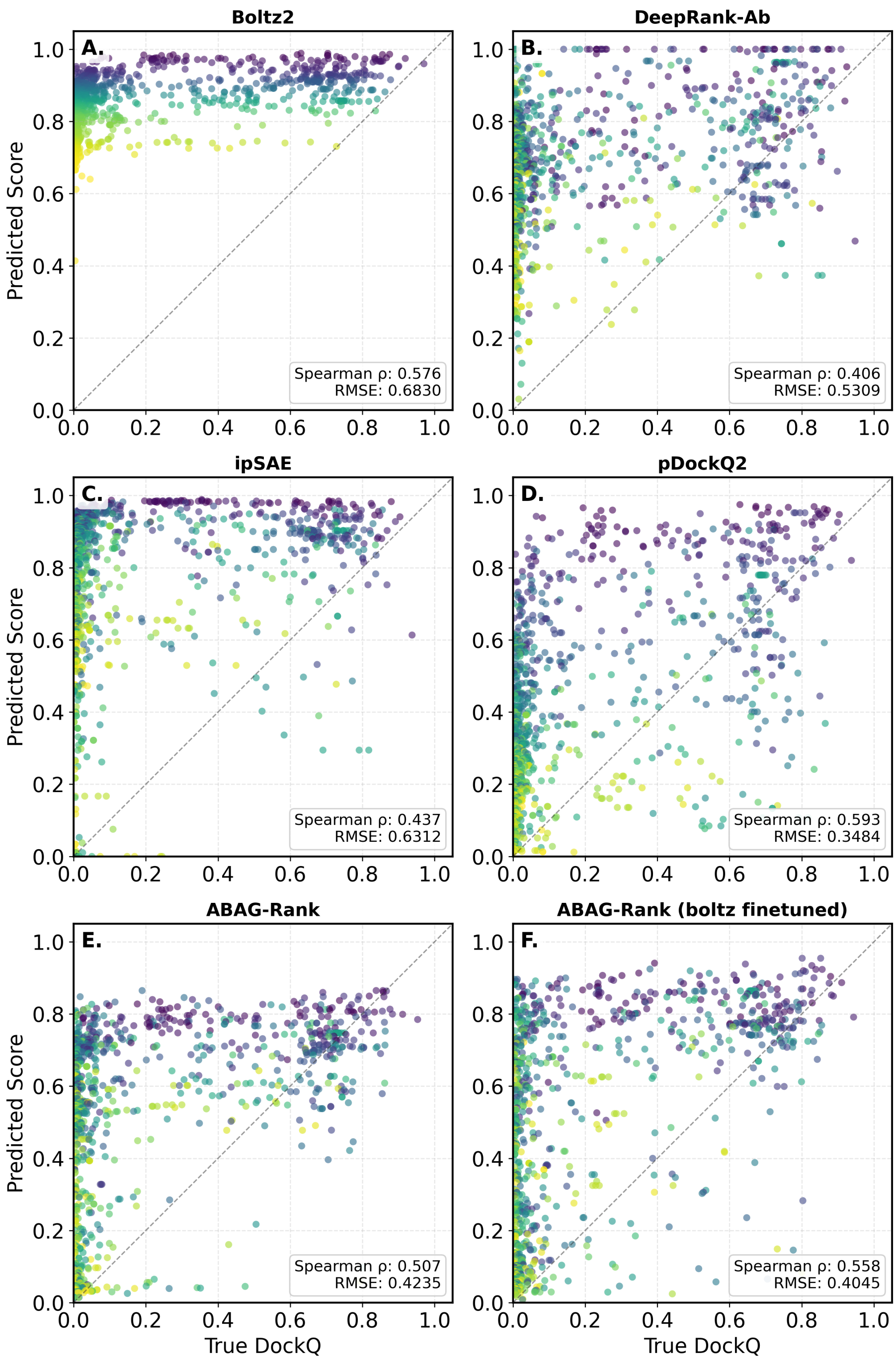

Fig. S12. Boltz2 scatter plot top-1: Comparison of ABAG-Rank and baseline models performance on the task of selecting the best-quality interface sample (top-1) on Boltz2 predictions

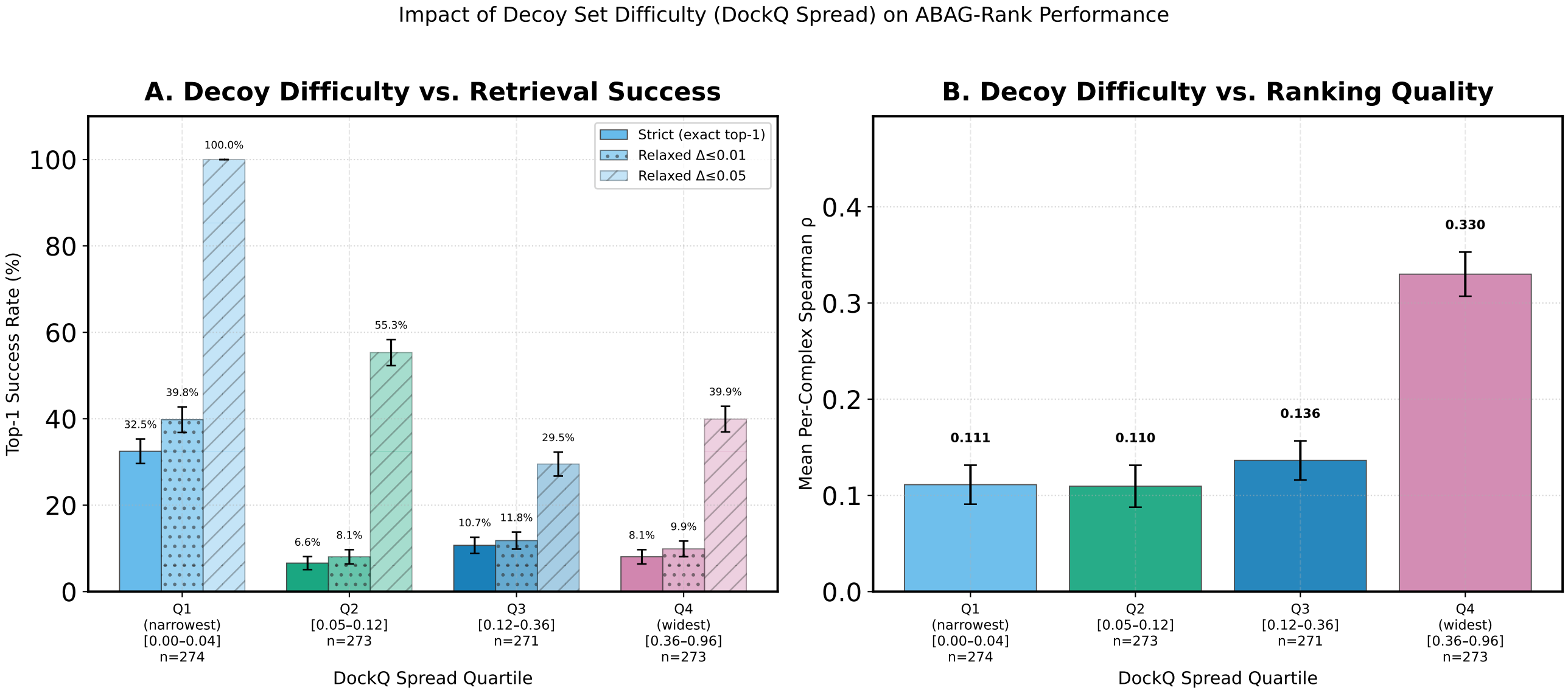

Fig. S13. Impact of Decoy-Set Difficulty on ABAG-Rank Performance. Complexes are binned into four quartiles of DockQ spread (max−min true DockQ per complex), from Q1 (narrowest spread, [0.00–0.04]) to Q4 (widest spread, [0.36–0.96]). **A.** Top-1 retrieval success rate under strict (exact top-1) and two relaxed ($\Delta \leq 0.01$, $\Delta \leq 0.05$) criteria. Q1 yields trivially high relaxed success (≈ 100%) because all decoys are nearly identical in quality, while strict success collapses for the middle quartiles. **B.** Mean per-complex Spearman ρ across quartiles. Ranking quality rises sharply for Q4, confirming that ABAG-Rank is most informative precisely when the landscape is diverse enough for ranking to matter.

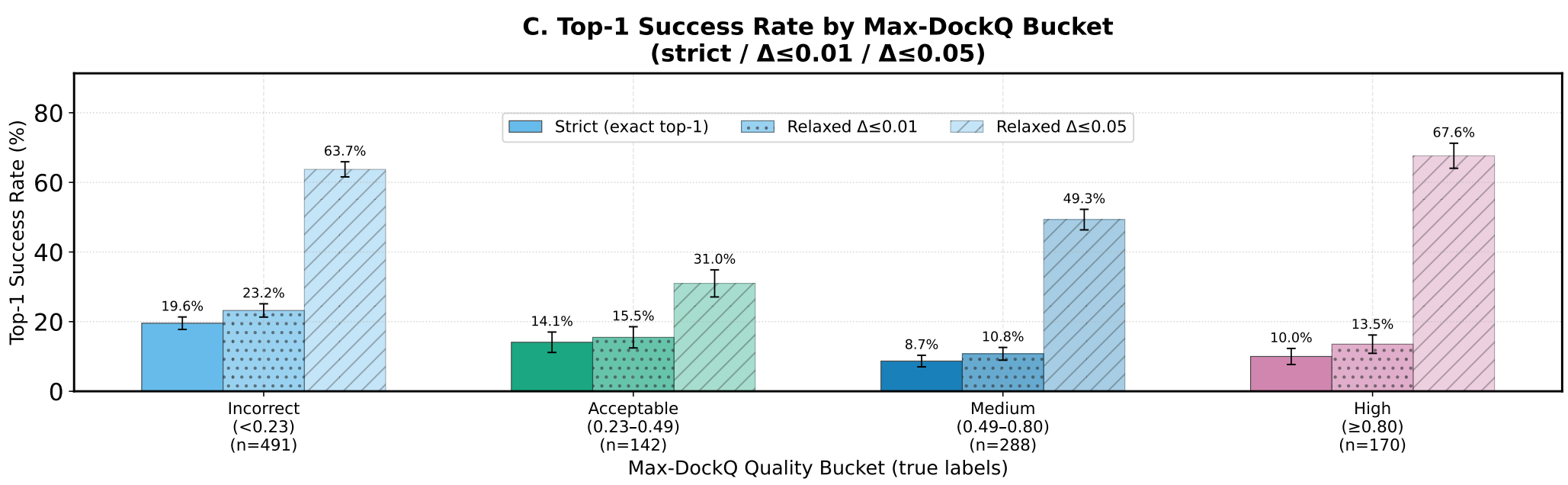

Fig. S14. Top-1 retrieval success rate under strict (exact top-1) and two relaxed ($\Delta \leq 0.01$, $\Delta \leq 0.05$) criteria for different DockQ thresholds.

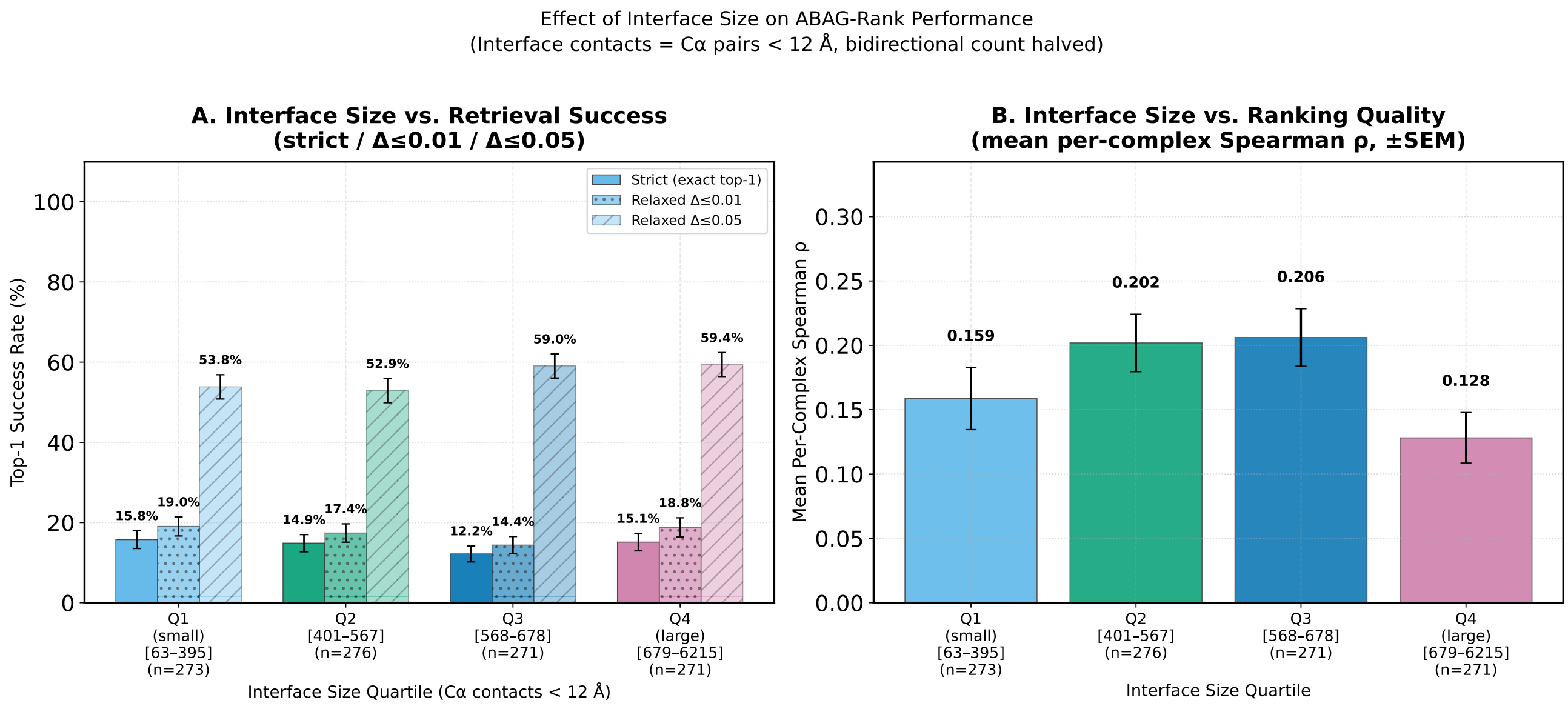

Fig. S15. Effect of Interface Size on ABAG-Rank Performance. Interface contacts are counted as Cα pairs within 12 ÅĊomplexes are split into four interface-size quartiles from Q1 (small, 63–395 contacts) to Q4 (large, 679–6215 contacts). **A.** Top-1 success rate under strict and relaxed criteria; success is broadly stable across sizes. **B.** Mean per-complex Spearman ρ (±SEM).

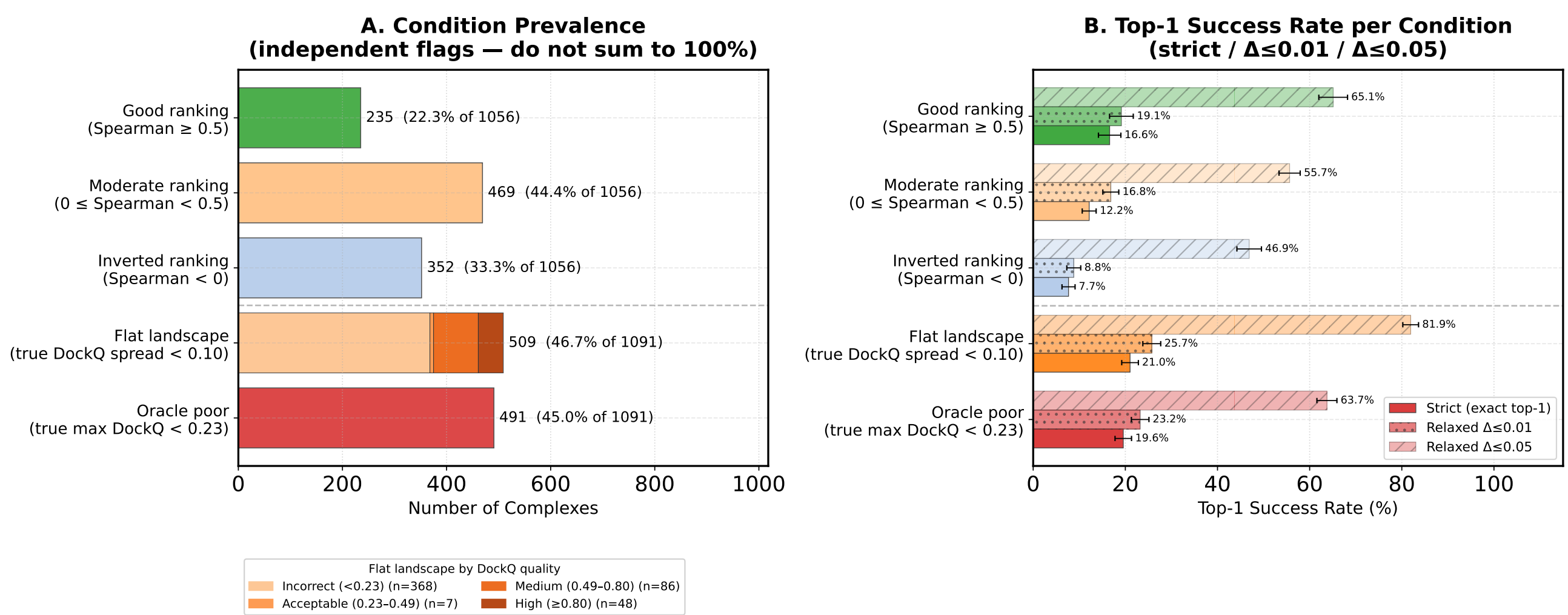

Fig. S16. Failure Condition Analysis for ABAG-Rank on the SAbDab Test Set. **A.** Prevalence of five conditions: *Oracle poor* (true max DockQ < 0.23, $n = 491$, 45.0%), *Flat landscape* (true DockQ spread < 0.10, $n = 509$, 46.7%), *Inverted ranking* (Spearman < 0, , 33.3%), *Moderate ranking* ($0 \leq \rho < 0.5$, 44.5%), and *Good ranking* ($\rho \geq 0.5$, 22.3%). **B.** Top-1 success rate per condition under strict and relaxed thresholds; complexes with good ranking reach 65.1% relaxed success. Notably, flat-landscape complexes still achieve 81.9% relaxed success because their decoys are intrinsically similar in quality.

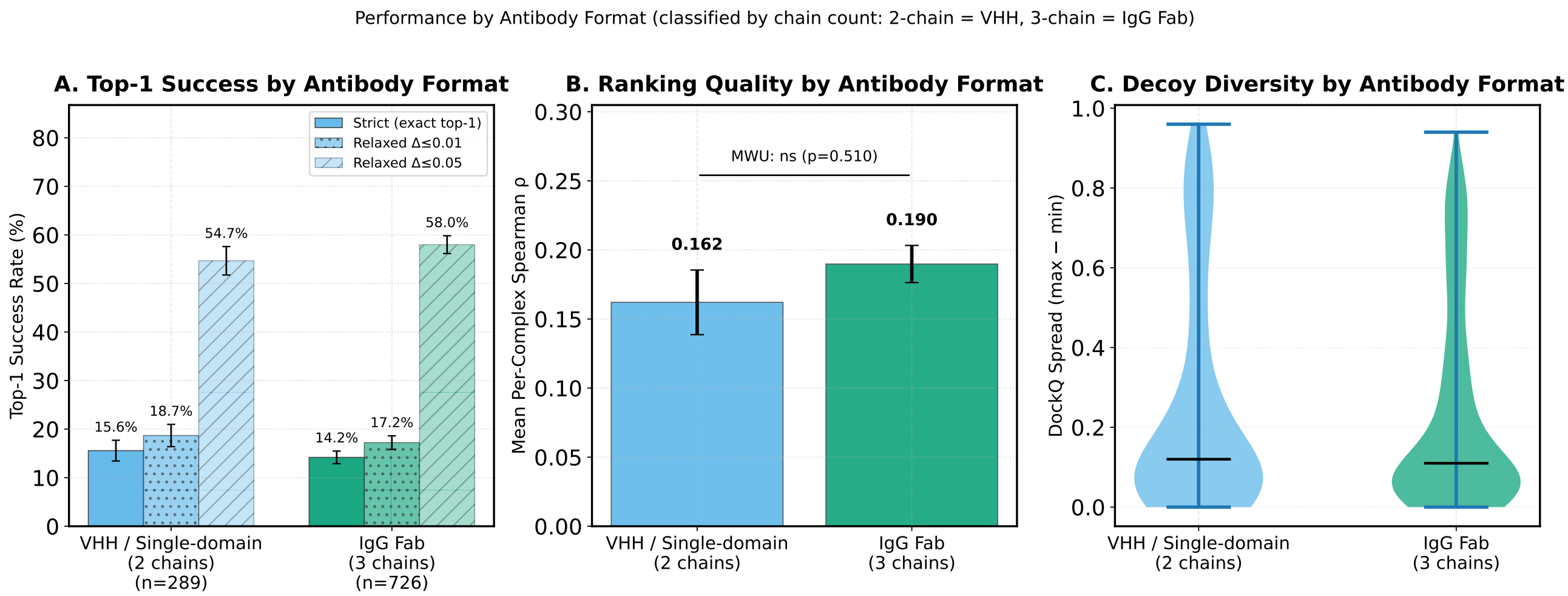

Fig. S17. Performance by Antibody Format (VHH/Single-domain vs. IgG Fab). **A.** Top-1 retrieval success rate for VHH/single-domain antibodies (2-chain, $n = 289$) and IgG Fab (3-chain, $n = 726$) under strict and relaxed criteria. **B.** Mean per-complex Spearman ρ (MWU test $p = 0.510$); the difference in ranking quality (0.190 vs. 0.162) is not statistically significant. **C.** Violin plots of DockQ spread per format.

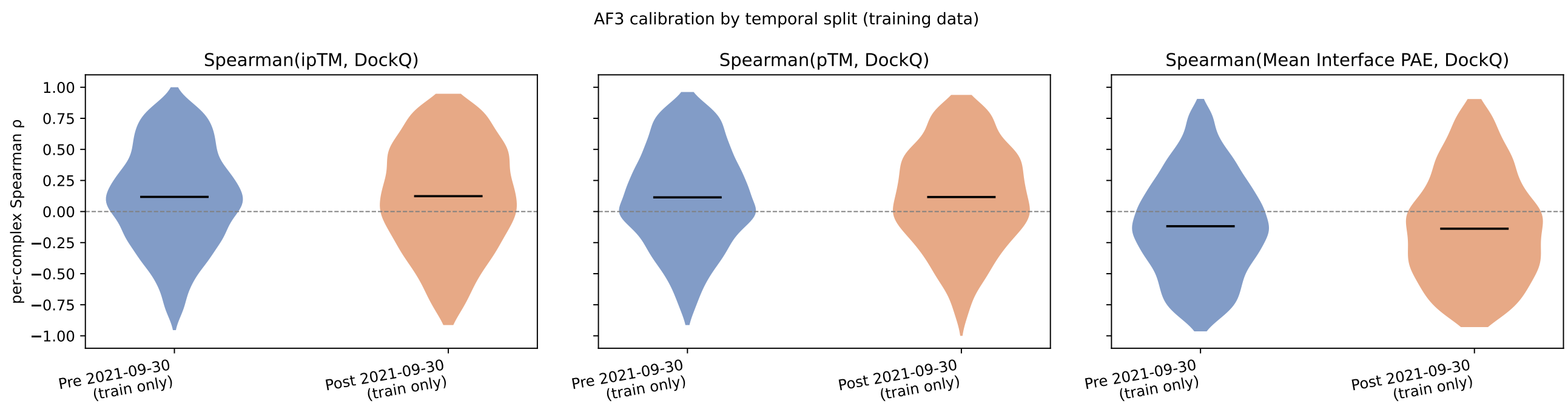

Fig. S18. Per-complex Spearman DockQ) distributions for training complexes deposited before and after the AF3 training cutoff (2021-09-30). Medians and shapes are indistinguishable, showing no evidence that AF3 memorisation inflates confidence calibration.

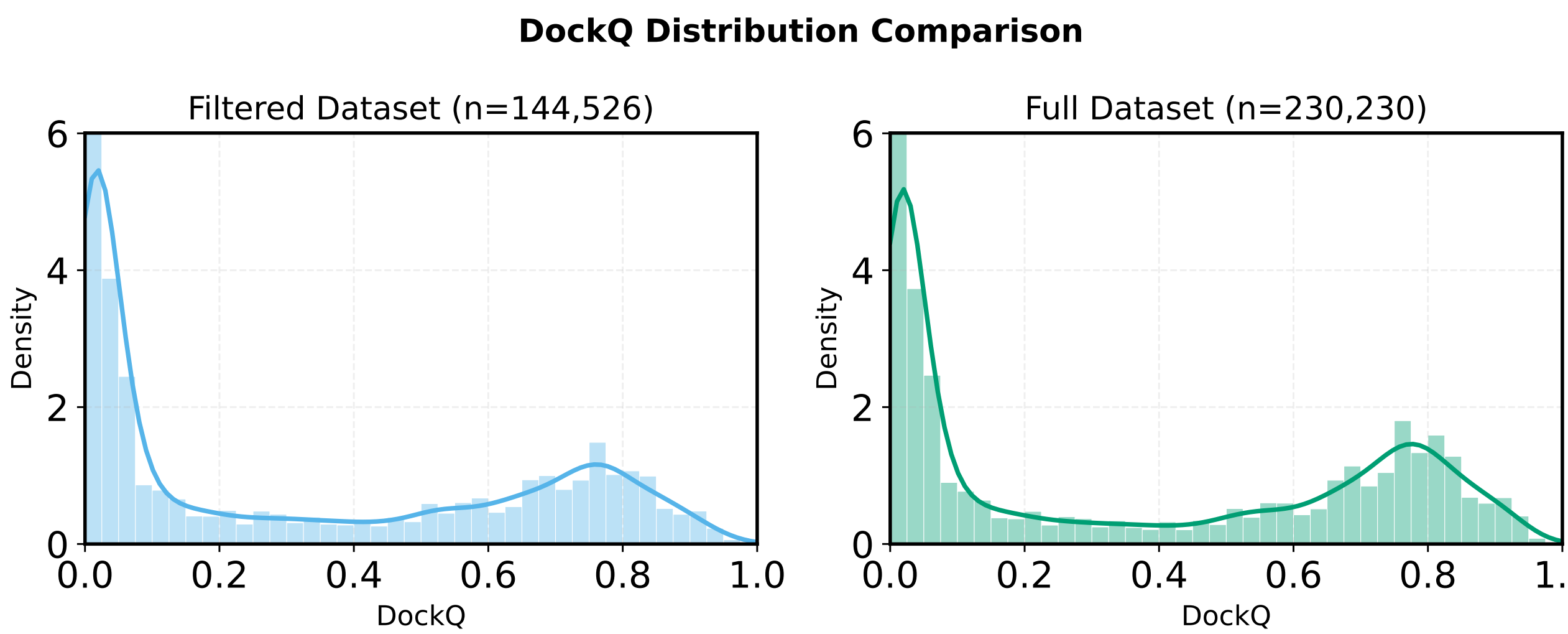

Fig. S19. DockQ distributions for training complexes before and after filtering; the filtering process maintains the same overall probability distribution by drastically reducing the number of samples necessary for training.

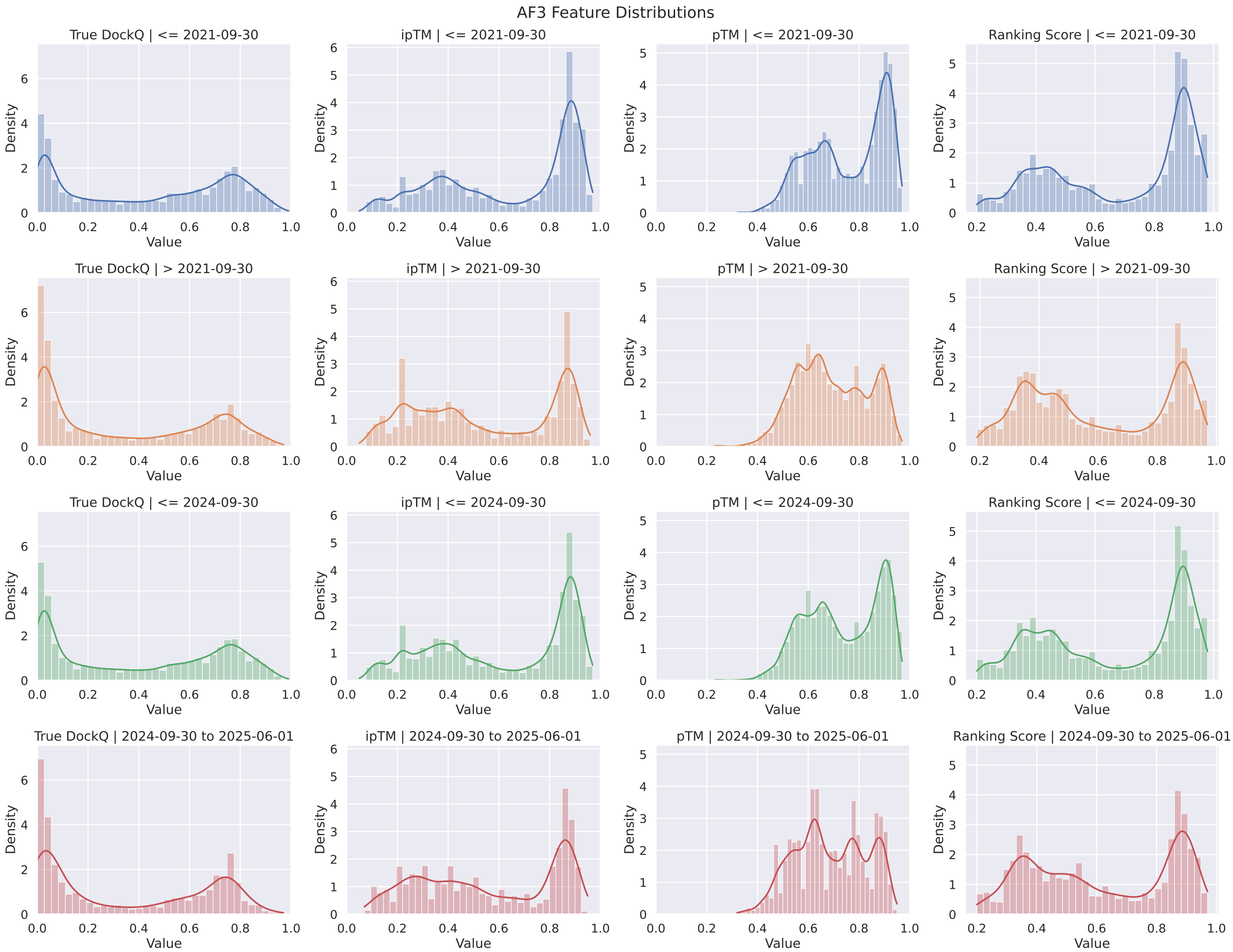

Fig. S20. An analysis of the various distributions of interface metrics from AF3 and the ground truth DockQ given the time split of AF3 training/test first two lines and ABAG-Rank second two lines. The distributions follow a very similar trend regardless of the time split, with a slightly higher number of incorrectly predicted interfaces for structures outside the AF3 training set, reinforcing the idea that AF3 has difficulty generating correct Ab-Ag complexes. The ABAG-Rank split allows us to have samples of AF3 both from their training set and new samples, allowing the model to have more complete input samples (both complexes with correct predictions and wrong predictions from AF3).

Supplementary Tables

Table S1. Maximum test-to-train sequence identity across resolution levels (n = 1,091 test complexes; training: SAbDab entries ≤ 2024-09-30, test: 2024-09-30 to 2025-06-01). CDR loops assigned via ANARCI (IMGT scheme, positions 27–38, 56–65, 105–117); pairwise identity computed with MMseqs2 (global end-to-end alignment, `--alignment-mode 3`, ≥80% bidirectional coverage, `--cov-mode 0 -c 0.8`), following the 80% redundancy threshold of Fromm et al. (2025). "No match" denotes test complexes with no training hit at ≥80% coverage. Heavy-chain CDR identity (median 0.689) and Ab-side interface identity (23.2% ≥80%) indicate that novelty is concentrated at the binding determinants, despite high framework/antigen conservation elsewhere; 56.4% of test epitopes have no training counterpart. On the resulting non-redundant subset (<80% CDR-H identity, N = 793), ranking performance slightly decreases (median Spearman ρ = 0.131 vs. 0.151 full set), but confirms generalisation rather than leakage.

Level	Median	≥80% (redundant)	No match
Full heavy chain	0.851	74.2%	0.1%
Full light chain	0.943	67.3%	30.9%
Antigen	0.997	56.2%	32.9%
CDR-H (H1+H2+H3)	0.689	27.3%	15.9%
CDR-L (L1+L2+L3)	0.909	51.7%	36.9%
Interface, Ab-side	0.722	23.2%	26.7%
Interface, Ag-side	0.861	29.0%	56.4%

Subset	N	ABAG-Rank ρ	AF3 baseline ρ
Full test set	1,091	0.151	0.106
Non-redundant (CDR-H <80%)	793	0.131	0.120

Table S2. Ranking performance by interface structural similarity to training set. TM≥0.6 (N=530 complexes) indicates interfaces structurally similar to training data; TM<0.6 (N=404 complexes) indicates novel interfaces. Statistical significance of per-complex Spearman against AlphaFold3: ns (p≥0.05), * (p<0.05), ** (p<0.01), *** (p<0.001). Bold indicates best performance in each metric.

**Method**	**RMSE**		**Global ρ**		**Per-Cplx ρ**	
	TM≥0.6	TM<0.6	TM≥0.6	TM<0.6	TM≥0.6	TM<0.6
AlphaFold3	0.3111	0.2662	0.779	0.562	0.134	**0.135**
DeepRank-Ab	0.2698	0.2618	0.691	0.542	0.129**	0.098***
ipSAE	0.4783	0.4942	0.558	0.310	0.002***	0.119***
pDockQ	0.3201	0.3476	0.541	0.213	0.108***	0.122***
pDockQ2	0.2020	0.1971	0.740	0.488	0.088***	0.109***
ABAG-Rank	**0.1684**	**0.1780**	**0.872**	**0.643**	**0.250*****	0.084***

Table S3. Performances for 500 samples per complex. Best performance is indicated as bold. RMSE: Root Mean Square Error, AUC: Area Under the Curve. Arrows down or up indicate the direction of preference.

	**Global Regression Metrics**			**AUC Classification (↑)**		
	**RMSE (↓)**	**Spearman (ρ) (↑)**		**Threshold (DockQ)**		
**Method**		**Global**	**Per Cplx**	**Acc** (> 0.23)	**Med** (> 0.50)	**High** (> 0.80)
Random	0.483	-0.002	-0.003	0.500	0.501	0.500
AlphaFold3	0.353	0.757	0.145	0.917	0.923	0.870
ABAG-Rank	**0.170**	**0.824**	**0.179**	**0.935**	**0.950**	**0.910**

Table S4. Performances for 500 samples per complex at selecting the best-interface quality Ab-Ag model among the top-K ranked samples. τ represents the true DockQ threshold. Best performance is indicated in bold.

	Top-K Success Rate (%) (↑)				Real Success Rate (%) (↑)					
	Acceptable (τ=0.23)		Medium (τ=0.50)		Best Decoy (Strict)		Relaxed Decoy ($\Delta \leq 0.01$)		Relaxed Decoy ($\Delta \leq 0.05$)	
**Method**	**T-1**	**T-5**	**T-1**	**T-5**	**T-1**	**T-5**	**T-1**	**T-5**	**T-1**	**T-5**
Oracle (Upper Bound)	71.19	71.19	50.00	50.00	100.00	100.00	100.00	100.00	100.00	100.00
Random	38.98	47.03	33.05	38.56	1.27	3.39	6.36	14.83	30.93	45.76
AlphaFold3	45.34	47.46	**38.56**	40.68	**2.97**	**8.47**	**8.05**	**21.61**	**40.68**	**50.85**
ABAG-Rank	**47.88**	**52.54**	36.86	**41.95**	2.12	**8.47**	**8.05**	18.22	35.59	50.00

Table S5. Ablation performance of ABAG-Rank at ranking Ab-Ag models according to their interface quality. Best performance is indicated as bold. RMSE: Root Mean Square Error, AUC: Area Under the Curve. Arrows down or up indicate the direction of preference.

	Global Regression Metrics			AUC Classification (↑) Threshold (DockQ)		
	RMSE (↓)	Spearman (ρ) (↑)				
**Method**		**Global**	**Per Cplx**	**Acc** (> 0.23)	**Med** (> 0.50)	**High** (> 0.80)
Random	0.489	-0.000	0.004	0.500	0.502	0.490
AlphaFold3	0.289	0.733	0.134	0.920	0.924	0.870
DeepRank-Ab	0.268	0.651	0.111^{**}	0.876	0.885	0.883
ABAG-Rank	**0.175**	**0.804**	$\mathbf{0.178}^{**}$	**0.935**	**0.946**	0.910
ABAG-Rank (unfiltered dataset)	0.204	0.758	0.142^{ns}	0.908	0.919	0.883
ABAG-Rank (without ESM)	0.189	0.786	0.163^{ns}	0.929	0.943	**0.918**
ABAG-Rank (without (i)ptm)	0.191	0.772	0.176^{**}	0.920	0.928	0.893
ABAG-Rank (without ESM, PAE)	0.195	0.761	0.169^{*}	0.926	0.937	0.880
ABAG-Rank (without ESM, PAE, (i)ptm)	0.284	0.641	0.139^{ns}	0.841	0.839	0.759

Table S6. Ablation performance of ABAG-Rank at selecting the best-interface quality Ab-Ag model among the top-K ranked samples. τ represents the true DockQ threshold. Best performance is indicated in bold.

	Top-K Success Rate (%) (↑)				Real Success Rate (%) (↑)					
	Acceptable (τ=0.23)		Medium (τ=0.50)		Best Decoy (Strict)		Relaxed Decoy ($\Delta \leq 0.01$)		Relaxed Decoy ($\Delta \leq 0.05$)	
**Method**	**T-1**	**T-5**	**T-1**	**T-5**	**T-1**	**T-5**	**T-1**	**T-5**	**T-1**	**T-5**
Oracle	58.77	58.77	44.80	44.80	100.00	100.00	100.00	100.00	100.00	100.00
Random	36.97	46.18	31.02	38.26	9.42	23.98	13.58	29.04	45.09	66.90
AlphaFold3	43.01	46.18	36.77	39.15	10.21	25.57	14.87	30.23	51.14	64.92
DeepRank-Ab	42.62	48.07	36.27	40.24	9.61^{ns}	25.87	14.57	30.92	51.54	69.08
ABAG-Rank	**44.90**	**49.26**	36.47	40.34	$\mathbf{13.08}^{*}$	**30.23**	**15.76**	**34.39**	54.11	**70.56**
ABAG-Rank (unfiltered dataset)	42.52	47.67	35.18	38.75	9.81^{ns}	27.65	12.49	30.92	50.05	67.89
ABAG-Rank (without ESM)	43.71	48.07	36.57	39.84	8.42^{ns}	25.57	11.89	30.03	48.66	67.20
ABAG-Rank (without (i)ptm)	43.21	48.46	36.37	39.64	11.89^{ns}	29.83	14.87	34.09	**54.41**	70.37
ABAG-Rank (without ESM, PAE)	43.81	47.77	**37.56**	**40.83**	8.52^{ns}	24.28	11.89	29.24	53.72	70.17
ABAG-Rank (without ESM, PAE, (i)ptm)	40.04	46.58	34.09	39.15	8.33^{ns}	23.59	11.20	27.55	47.47	66.40

Table S7. Performance on Boltz2 predictions: Performance of ABAG-Rank and baselines at ranking Ab-Ag models according to their interface quality. Best performance is indicated as bold. RMSE: Root Mean Square Error, AUC: Area Under the Curve. Arrows down or up indicate the direction of preference. Statistical significance is denoted by ^{ns} (not significant), * ($p < 0.05$), and *** ($p < 0.001$). † for DeepRank-Ab denotes default inference settings of the published model.

	Global Regression Metrics			AUC Classification (↑) Threshold (DockQ)		
	RMSE (↓)	Spearman (ρ) (↑)				
**Method**		**Global**	**Per Cplx**	**Acc** (> 0.23)	**Med** (> 0.50)	**High** (> 0.80)
Random	0.488	0.007	0.005	0.503	0.500	0.514
Boltz2	0.646	0.626	**0.095**	0.822	0.822	0.835
DeepRank-Ab	0.313	0.536	0.091^{ns}	0.803	0.819	**0.860**
ipSAE	0.542	0.473	0.033***	0.714	0.689	0.643
pDockQ2	0.270	0.578	0.084^{ns}	0.800	0.785	0.835
ABAG-Rank	0.289	0.613	0.087^{ns}	0.840	0.815	0.837
ABAG-Rank (boltz finetuned)	**0.262**	**0.664**	0.072*	**0.859**	**0.844**	0.830

Table S8. Performance on Boltz2 predictions: Performance of ABAG-Rank and baselines at selecting the best-interface quality Ab-Ag model among the top-K ranked samples. τ represents the true DockQ threshold. Best performance is indicated in bold. Statistical significance against baseline is denoted by ^{ns} (not significant), * ($p < 0.05$), ** ($p < 0.01$), and *** ($p < 0.001$).

	Top-K Success Rate (%) (↑)				Real Success Rate (%) (↑)					
	**Acceptable** (τ=0.23)		**Medium** (τ=0.50)		**Best Decoy** (Strict)		**Relaxed Decoy** ($\Delta \leq 0.01$)		**Relaxed Decoy** ($\Delta \leq 0.05$)	
**Method**	**T-1**	**T-5**	**T-1**	**T-5**	**T-1**	**T-5**	**T-1**	**T-5**	**T-1**	**T-5**
Oracle	47.66	47.66	32.47	32.47	100.00	100.00	100.00	100.00	100.00	100.00
Random	31.63	39.54	21.33	26.01	3.85	14.05	19.67	38.09	50.99	70.66
Boltz2	34.03	39.44	23.20	**28.20**	5.83	17.69	21.96	40.89	54.32	74.30
DeepRank-Ab	**35.38**	**40.79**	**24.56**	27.99	**6.76**^{ns}	**19.15**	**24.25**	**42.04**	**54.94**	**74.40**
ipSAE	33.09	40.69	22.48	27.99	5.10^{ns}	18.00	20.60	39.33	52.55	73.88
pDockQ2	34.13	39.54	23.52	27.47	5.93^{ns}	16.96	22.06	40.58	51.61	73.78
ABAG-Rank	34.44	39.85	23.83	27.68	5.31^{ns}	18.63	21.96	41.83	53.38	73.88
ABAG-Rank (boltz finetuned)	34.24	39.75	22.79	26.85	5.52^{ns}	17.17	21.44	38.81	52.76	72.32

Dataset Filtering Algorithms

Algorithm 1 IsRedundant

Require: sample s, kept sample k, thresholds τ_{DockQ}, $\tau_{\text{PAE}}, \tau_{\text{RMSD}}$
Ensure: **true** iff s is redundant w.r.t. k

$\Delta_{\text{DockQ}} \leftarrow |\text{dockq}(s) - \text{dockq}(k)|$
if $\Delta_{\text{DockQ}} > \tau_{\text{DockQ}}$ **then**
    return false ▷ Distinct quality, keep sample
$\Delta_{\text{PAE}} \leftarrow \text{Wasserstein}_1\big(\text{pae}(s), \text{pae}(k)\big)$
if $\Delta_{\text{PAE}} \geq \tau_{\text{PAE}}$ **then**
    return false ▷ Different confidence profile, keep sample
$\Delta_{\text{RMSD}} \leftarrow \text{rmsd}\big(\text{CA}(s), \text{CA}(k)\big)$
if $\Delta_{\text{RMSD}} \geq \tau_{\text{RMSD}}$ **then**
    return false ▷ Structurally distinct, keep sample
return true ▷ Similar DockQ, PAE, and structure, redundant

Algorithm 2 GreedyPrune

Require: ordered list $\mathcal{S}$, thresholds τ_{DockQ}, τ_{PAE}, τ_{RMSD}
Ensure: sets $\mathcal{K}$ (kept), $\mathcal{P}$ (pruned), clustering map $\mathcal{C}$

$\mathcal{K} \leftarrow \{\mathcal{S}_0\}; \quad \mathcal{P} \leftarrow \varnothing; \quad \mathcal{C}[\mathcal{S}_0] \leftarrow \varnothing$
for each $s \in \mathcal{S} \setminus \{\mathcal{S}_0\}$ **do**
    is_redundant $\leftarrow$ **false**
    for each $k \in \mathcal{K}$ **do**
        if IsRedundant(s, k, $\tau_{\text{DockQ}}, \tau_{\text{PAE}}, \tau_{\text{RMSD}}$) **then**
            is_redundant $\leftarrow$ **true**
            $\mathcal{P} \leftarrow \mathcal{P} \cup \{s\}$
            $\mathcal{C}[k] \leftarrow \mathcal{C}[k] \cup \{(s, \Delta_{\text{DockQ}}, \Delta_{\text{PAE}}, \Delta_{\text{RMSD}})\}$
            break
    if *is_redundant* $=$ **false then**
        $\mathcal{K} \leftarrow \mathcal{K} \cup \{s\}; \quad \mathcal{C}[s] \leftarrow \varnothing$
return $(\mathcal{K}, \mathcal{P}, \mathcal{C})$
